# Effectiveness and cost-effectiveness of universal school-based mindfulness training compared with normal school provision in reducing risk of mental health problems and promoting well-being in adolescence: the MYRIAD cluster randomised controlled trial

**DOI:** 10.1136/ebmental-2021-300396

**Published:** 2022-07-07

**Authors:** Willem Kuyken, Susan Ball, Catherine Crane, Poushali Ganguli, Benjamin Jones, Jesus Montero-Marin, Elizabeth Nuthall, Anam Raja, Laura Taylor, Kate Tudor, Russell M Viner, Matthew Allwood, Louise Aukland, Darren Dunning, Tríona Casey, Nicola Dalrymple, Katherine De Wilde, Eleanor-Rose Farley, Jennifer Harper, Nils Kappelmann, Maria Kempnich, Liz Lord, Emma Medlicott, Lucy Palmer, Ariane Petit, Alice Philips, Isobel Pryor-Nitsch, Lucy Radley, Anna Sonley, Jem Shackleford, Alice Tickell, Sarah-Jayne Blakemore, The MYRIAD Team, Obioha C Ukoumunne, Mark T Greenberg, Tamsin Ford, Tim Dalgleish, Sarah Byford, J Mark G Williams

**Affiliations:** 1 Department of Psychiatry, Warneford Hospital, University of Oxford, Oxford, UK; 2 NIHR Applied Research Collaboration South West Peninsula (PenARC), University of Exeter, Exeter, Devon, UK; 3 King’s College London, King’s Health Economics, Institute of Psychiatry, Psychology and Neuroscience, De Crespigny Park, London, UK; 4 Teaching, Research and Innovation Unit, Parc Sanitari Sant Joan de Déu, Sant Boi de Llobregat, Spain; 5 Population, Policy & Practice research programme, UCL Great Ormond St. Institute of Child Health, London, UK; 6 Medical Research Council Cognition and Brain Sciences Unit, University of Cambridge, Cambridge, UK; 7 Department of Psychology, University of Cambridge, Cambridge, UK; 8 UCL Institute of Cognitive Neuroscience, London, UK; 9 Department of Human Development and Family Studies, The Pennsylvania State University, University Park, Pennsylvania, USA; 10 Department of Psychiatry, University of Cambridge, Cambridge Biomedical Campus, Cambridge, UK

**Keywords:** Child & adolescent psychiatry, Depression & mood disorders

## Abstract

**Background:**

Systematic reviews suggest school-based mindfulness training (SBMT) shows promise in promoting student mental health.

**Objective:**

The My Resilience in Adolescence (MYRIAD) Trial evaluated the effectiveness and cost-effectiveness of SBMT compared with teaching-as-usual (TAU).

**Methods:**

MYRIAD was a parallel group, cluster-randomised controlled trial. Eighty-five eligible schools consented and were randomised 1:1 to TAU (43 schools, 4232 students) or SBMT (42 schools, 4144 students), stratified by school size, quality, type, deprivation and region. Schools and students (mean (SD); age range=12.2 (0.6); 11–14 years) were broadly UK population-representative. Forty-three schools (n=3678 pupils; 86.9%) delivering SBMT, and 41 schools (n=3572; 86.2%) delivering TAU, provided primary end-point data. SBMT comprised 10 lessons of psychoeducation and mindfulness practices. TAU comprised standard social-emotional teaching. Participant-level risk for depression, social-emotional-behavioural functioning and well-being at 1 year follow-up were the co-primary outcomes. Secondary and economic outcomes were included.

**Findings:**

Analysis of 84 schools (n=8376 participants) found no evidence that SBMT was superior to TAU at 1 year. Standardised mean differences (intervention minus control) were: 0.005 (95% CI −0.05 to 0.06) for risk for depression; 0.02 (−0.02 to 0.07) for social-emotional-behavioural functioning; and 0.02 (−0.03 to 0.07) for well-being. SBMT had a high probability of cost-effectiveness (83%) at a willingness-to-pay threshold of £20 000 per quality-adjusted life year. No intervention-related adverse events were observed.

**Conclusions:**

Findings do not support the superiority of SBMT over TAU in promoting mental health in adolescence.

**Clinical implications:**

There is need to ask what works, for whom and how, as well as considering key contextual and implementation factors.

**Trial registration:**

Current controlled trials ISRCTN86619085. This research was funded by the Wellcome Trust (WT104908/Z/14/Z and WT107496/Z/15/Z).

**WHAT IS ALREADY KNOWN ON THIS TOPIC**

- Prior to the My Resilience in Adolescence (MYRIAD) Trial, comprehensive systematic reviews have suggested evidence of small effects of school-based mindfulness training (SBMT) on mental health outcomes and well-being.
- However, studies have tended to be small, with short or no follow-ups, marked heterogeneity in terms of outcomes, control conditions, and target populations, as well as evidence of possible publication bias.

**WHAT THIS STUDY ADDS**

- Using an adequately powered randomised controlled trial design, with a 1 year follow-up, the MYRIAD Trial found no such evidence of superiority of universal SBMT over normal provision of social-emotional education, but some evidence of cost-effectiveness in terms of quality-adjusted life years.

**HOW THIS STUDY MIGHT AFFECT RESEARCH, PRACTICE AND/OR POLICY**

- The next generation of research needs to consider what works, for whom and how, as well as considering key contextual and implementation factors.

## Introduction

Mental health problems commonly have their first onset in adolescence.^1^ Of all mental health disorders that emerge during adolescence, depression has the largest impact on health throughout the life span in terms of years lost to disability.^2^ Preadult onset is associated with poorer lifetime mental health outcomes, greater impairments in social, emotional and occupational functioning, and reduced quality of life. Furthermore, broader social, emotional and behavioural problems often appear in this same developmental window, and predict mental health outcomes into adulthood.^3^ There have been many calls to develop programmes for adolescents to reduce risk of mental ill-health and promote well-being.^3^ Because schools play a central role in the lives of children and families, they provide an opportune setting for promoting mental health and preventing mental health problems.^4^

Systematic reviews suggest that school-based universal approaches to social-emotional learning, offered to a whole population, have the most potential to cost-effectively promote the mental health and well-being of young people.^4^ Our premise was that skills in attention and social-emotional-behavioural self-regulation underpin mental health and well-being across the full spectrum of well-being. As such, we suggest the need for a training method that focuses on teaching these skills, instead of focusing on reducing pathology-specific patterns of negative thinking and unhelpful behaviour. Our programme aims to examine one such method, school-based mindfulness training (SBMT), which is specifically designed to address those processes and can be used by young people across the spectrum of mental health. Our pilot work,^5^ a systematic review^6^ and a scoping review^7^ suggested SBMT can be acceptable and shows promise of effectiveness, but requires attention to implementation. Considering all of this, the study’s primary aim was to determine the effectiveness and cost-effectiveness of a universal SBMT programme delivered by school teachers and designed to be used by young people aged 11–16 years across the spectrum of mental health. A cluster (school) randomised controlled trial evaluated the effectiveness of SBMT compared with teaching as usual (TAU) on three co-primary outcomes (eg, risk for depression, social-emotional-behavioural functioning and well-being) at 1 year follow-up, measured at the level of the individual young person.

## Method

The trial is reported in accordance with Consolidated Standards of Reporting Trials (CONSORT) guidelines for cluster randomised controlled trials.^8^ The study design and procedures are presented in full in the published trial protocol^9^ and update describing study enhancements and adaptations necessitated by the COVID-19 pandemic.^10^ The main change was establishing the 1 year follow-up, as opposed to the 2 years follow-up, as the primary end point to ensure all the outcome data were collected prior to the pandemic. Other changes included an elaboration of the school eligibility criteria, a greater number of schools and students that had to be recruited, and some changes in the secondary outcomes. These changes were considered through learning in the conduct of the trial. They facilitated a clearer definition to assess Social Emotional Learning (SEL) implementation in schools, prevented contamination across trial arms, allowed us to recruit extra schools and students to cope with dropout at the school and student levels, and enabled us to improve the validity of some of our secondary outcomes.^10^

### Study design and participants

The ‘MYRIAD: My Resilience in Adolescence, a study examining the effectiveness and cost-effectiveness of a mindfulness training programme in schools compared with normal school provision’ includes a superiority cluster randomised controlled parallel group trial (registration number ISRCTN86619085). The trial examined the effectiveness of inclusion of the SBMT programme within school social-emotional teaching provision compared with provision of school social-emotional TAU on any or all of three co-primary outcomes. Because SBMT is a universal, school-based intervention, we used a cluster trial design, with schools as the unit of randomisation. Classes were subsampled within schools and then all children in those classes were recruited by complete enumeration.

**Figure 1:**
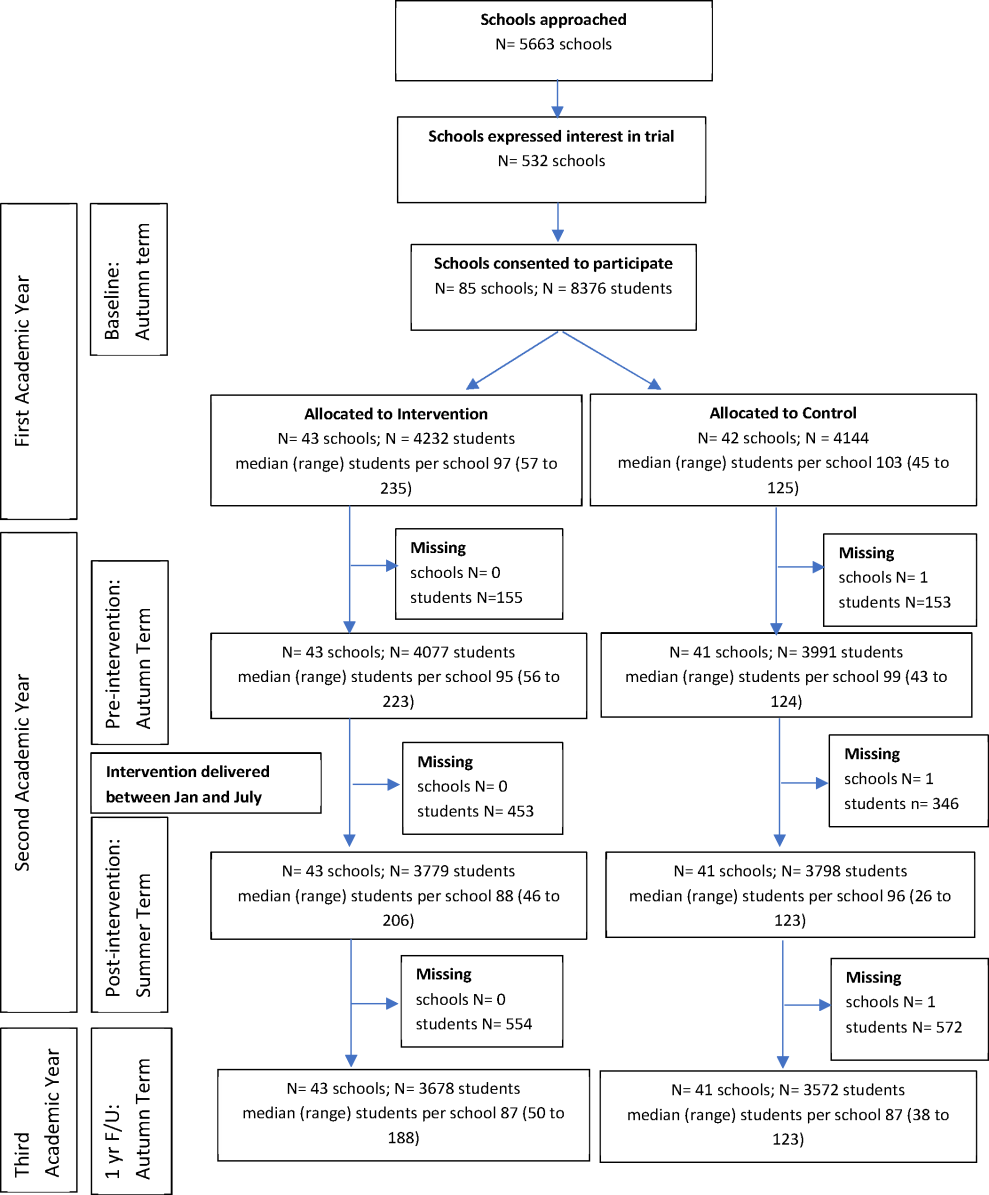
Consort flow diagram for trial. ‘Missing’ is the number of schools (N)/students (N) that did not provide data on any of the three primary outcomes at the subsequent time point. Students could be temporarily lost to follow-up if not in school for a given time point. For the teaching-as-usual (TAU) school that dropped out soon after randomisation, no pupils were included in the trial and so no data were provided.

Participant flow is described in the study CONSORT flow chart (figure 1). All mainstream secondary schools in the UK, including private schools, were eligible if they had a substantive appointed headteacher, had not been judged inadequate in their most recent official inspection (to mitigate any risk of difficulties in trial implementation), and had a strategy and structure in place for delivery of adequate SEL curricula. Recruitment was conducted in two cohorts (academic years 2016/2017 and 2017/2018), and involved first consenting schools, then providing parents an option to opt their children out, then assenting the young people themselves. Due to the nature of SBMT, students and staff were not blind to treatment allocation.

### Setting

Participating schools were broadly representative of UK secondary schools with respect to the population served on key variables such as deprivation, operationalised as the percentage of children eligible for free school meals, and the type of school (ie, selective/non-selective, urban/rural, large/small, mixed/single gender, state maintained/independent). All offered social and emotional teaching in line with good practice guidance (see online supplemental F).^11^

10.1136/ebmental-2021-300396.supp1 Supplementary data

### Randomisation and allocation concealment

Following collection of baseline data, schools were randomised in a 1:1 ratio to trial arms by an independent researcher otherwise unconnected with the trial. The independent statistician generated the allocation sequence, and the trial manager enrolled the clusters and assigned them to the trial arms. For both cohorts, allocation of schools was balanced on: school size (large (1000 children or more) vs small (fewer than 1000 children)); school quality (‘outstanding/good’ vs ‘requires improvement’); and level of deprivation (below vs above the median percentage across all UK schools of children eligible for free school meals—29.4% based on UK Department of Education data in 2017). In addition, for Cohort 2, allocation was also balanced by type of school (boys, girls and mixed) and region (England, Scotland, Wales and northern Ireland). A constrained randomisation approach^12^ was used, where the allocation sequence was selected to achieve a high level of balance on the above factors between the intervention and control arms. Allocation concealment (cluster level) was achieved as all schools were recruited before randomisation and allocated en bloc for each cohort.^9 10^ We avoided recruitment bias by recruiting students (individual participant level, age: mean 12.2, range 10–14 years) before the schools were randomised.^13^

### Interventions

The SBMT programme and TAU are described fully in the protocol and in the online supplemental materials 9 (see online supplemental A). The intervention relates to the cluster level.

#### SBMT programme

Mindfulness is a natural and trainable capacity to bring awareness to both inner (eg, thoughts, feelings, body sensations) and outer (eg, stressors, relationships) experiences with qualities of curiosity, kindness and responsiveness. The SBMT programme aims to teach mindfulness skills that support young people’s resilience, using a combination of psychoeducation, class discussion and brief mindfulness practices. It was adapted from mindfulness-based cognitive therapy to make it acceptable to young people across the full spectrum of functioning from mental health problems to flourishing. It is integrated into the school curriculum and taught by school teachers. The SBMT involves 10 manualised, structured lessons (typically 30–50 min each), normally delivered over one school term (either in the first or second year of secondary school). The intention is to introduce young people to a range of skills (eg, attentional control, self-regulation of thoughts, feelings and behaviours) and they were encouraged to use these in their everyday lives. There are resources to support SBMT teachers (course booklets) and worksheets and online mindfulness practices for students. Because implementation affects both reach and outcomes,^14^ all schools allocated to SBMT were supported through: information provision to school leadership teams, SBMT teacher selection and training, and support of at least one round of teaching the MT curriculum prior to teaching study students (see online supplemental A). The SBMT teacher training and SBMT programme were optimised to maximise fidelity based on a previous work.^15^ All SBMT classes in the trial were recorded and a randomly selected subset of classes rated for fidelity by independent assessors using the Mindfulness Based Interventions – Teacher Assessment Criteria, adapted for school settings (see online supplemental B, Teacher Measures).

#### Teaching as usual

The aim of the trial was to establish if SBMT adds value to current good SEL practice in secondary schools. SEL provision aims to prepare students with the knowledge, skills and attributes they need to manage their lives. It typically covers relationships, sex education, and physical and mental health education. SEL provision was assessed using a bespoke tool based in part on existing measures. All control schools were offering some degree of structured SEL provision (mean (SD): 12.0 (2.6); possible range 0–16) (see online supplemental B, School Measures).

### Outcome measures

Study outcomes were measured at the individual (student) level at school consent/baseline (prior to randomisation), preintervention, postintervention (or equivalent time in the TAU arm) and at 1 year follow-up (1 year after preintervention measures). Students typically took a single lesson (<50 min) to complete the measures, and we prioritised completion of primary and cost-effectiveness measures over secondary outcomes.

*Co-primary outcome measures* were: self-reported risk for depression (Center for Epidemiologic Studies for Depression Scale; CES-D);^16^ self-reported social-emotional behavioural functioning (Strengths and Difficulties Questionnaire, SDQ, Youth Self-Report Version, total difficulties score);^17^ and self-reported well-being (Warwick-Edinburgh Mental Well-being Scale; WEMWBS)^18^ at the 1 year follow-up primary time point.

*Secondary outcome measures* at 1 year follow-up and postintervention were: students’ executive function (Behaviour Rating Inventory of Executive Function, Second Edition, self and teacher-rated versions, BRIEF-2); self-reported drug and alcohol use (measure designed for study); self-reported anxiety (anxiety subscales from the Revised Child Anxiety and Depression Scale, RCADS); teacher-reported social-emotional-behavioural functioning (SDQ, teacher version); self-reported self-harm and suicidal ideation (measures devised for study), school climate (subscales from the School Climate and Connectedness Survey, SCCS) and self-reported mindfulness skills (Child and Adolescent Mindfulness Measure, CAMM). Risk for depression, social-emotional-behavioural functioning and well-being postintervention were also secondary outcomes.

In the SBMT arm only, students rated the acceptability of and their engagement with SBMT (home-based practice) using bespoke measures.

For a full description of all measures see online supplemental B.

#### Economic data

We used the Child Health Utility 9D (CHU9D) Index measure of health-related quality of life.^19 20^ This is suitable for the calculation of quality-adjusted life years (QALYs) and has been shown to be valid and responsive to change in adolescent populations.

The economic evaluation took a health and social care perspective, as preferred by the National Institute for Health and Care Excellence, but additionally included education-based services, since evidence suggests that health and education make up the majority of the costs of caring for young people with depression.^21^ Service use was recorded using a brief version of the Child and Adolescent Service Use Schedule (CA-SUS), and was based on versions of the CA-SUS applied in adolescent depression populations.^21^ It focuses on key areas of service use (high cost and/or high volume of use), including hospital contacts (admissions and appointments, accident and emergency and ambulance), various community and school-based health services, medication for mental health conditions, teaching support, and accommodation (respite, residential or foster care). Young people were asked if they had made use of each service and follow-up questions to indicate amount of use were asked only where students indicate they had. It was designed for completion by parents of primary school children in a school-based cluster randomised controlled trial,^22^ and we adapted it for self-completion in this study. Economic data were collected preintervention and postintervention and at 1 year follow-up. At each assessment time point the measure collected information covering the previous 3 months.

Resource inputs into SBMT training and delivery were recorded as part of the trial and costed using a micro-costing approach. All other services used were costed by applying nationally applicable unit costs (see online supplemental C).

### Statistical analysis

The analysis followed a prespecified analysis plan. The target sample size of 84 schools and 6300 students (42 schools and 3150 students in each trial arm) was calculated to detect a difference of 0.2 SD units (effect size) on our three continuous co-primary outcomes with 90% power at the 5% (two-sided) level of significance.^9^ The following assumptions were made: three classes participating in each school; 25 of the 30 students in each class consenting to participate; 10% loss to follow-up at the school level and 20% loss to follow-up at the student level; and an intracluster (intraschool) correlation coefficient of 0.04 (the largest intracluster (intraschool) correlation coefficient in one of our feasibility studies was 0.037).^5^ The sample size also allows for multiple testing adjusting for three co-primary outcomes, based on the Bonferroni procedure, setting the two-tailed significance level (alpha) for comparing each individual outcome between the trial arms to 0.0167 to preserve the overall familywise type I error rate at 0.05. The sample size calculation assumes equal-sized clusters as we found that allowing for anticipated variation in cluster size made little difference.

Characteristics were summarised using means and SD for continuous variables and numbers and percentages for categorical variables. Outcomes were compared between the trial arms using the intention-to-treat principle, with participants analysed according to their trial arm. The total score for participants missing a subset of <20% of items for a given measure were ‘scaled-up’ based on the average score across non-missing items, unless the relevant scoring manuals suggested an alternative approach for management of missing items. Missing outcome data (assumed to be missing at random) were imputed using the multivariate linear mixed effects (‘multilevel’) model. The imputation model included the primary and secondary outcomes, trial arm status, costs, a measure of utility (CHU9D), stratification factors and other characteristics prespecified for covariate adjustment, and an auxiliary variable of the number of intervention sessions attended, generating 20 imputed data sets. In the main results tables, means and SD based on analyses of complete case data are reported alongside the findings of between-group analyses of the imputed data sets. Sensitivity analyses of the modified intention-to-treat population (ie, using complete case data only) were also undertaken for each primary and secondary outcome. Continuous outcomes were compared using mixed effects linear regression models and binary outcomes were compared using marginal logistic regression models using generalised estimating equations with information sandwich (‘robust’) estimates of SE; these methods allowed for correlation between observations from the same school (cluster), and the mixed effect linear regression of the continuous outcomes also allowed for an intermediate level of clustering at the class level. The outcomes were adjusted for the factors used to balance randomisation, cohort, student gender and baseline score on the outcome. Estimates of the intracluster (intraschool) correlation coefficients are reported, based on crude (unadjusted) analyses of outcome data. Tests of interaction were used to examine whether there is evidence that the intervention effect differs between Cohorts 1 and 2.

Cost-effectiveness was primarily assessed in terms of QALYs derived from the CHU9D in a cost-utility analysis.^20^ Secondary analyses explored cost-effectiveness in terms of co-primary outcomes to assess the sensitivity of analyses to the alternative outcomes of interest. Differences in costs and outcomes between trial arms were assessed using standard parametrical tests. Joint effects of costs and outcomes (QALYS or co-primary outcomes, as relevant) were estimated using mixed effects linear regression. Cost-utility and cost-effectiveness was assessed using the net benefit approach,^23^ with uncertainty explored through the presentation of cost-effectiveness acceptability curves.^24^ Prespecified sensitivity analyses assessed the impact of missing data (complete case analysis) and of different perspectives (excluding teaching support for a strict health and social services perspective and a narrower focus on mental health services only) . For full details of economic methods see online supplemental D.

Data for both the effectiveness and cost-effectiveness analyses were imputed using the *pan* package in R V.3.6.1 software,^25^ and analysed using the *mi* suite of commands in Stata V.16.1 software.

## Results

Recruitment is summarised in the CONSORT flow diagram (figure 1; for further details see online supplemental E). We recruited schools (n=85 clusters) in the academic years 2016/2017 (Cohort 1; n=13) and 2017/2018 (Cohort 2; n=72); 43 were randomised to SBMT and 42 to TAU. One school in Cohort 1 in the TAU arm withdrew from the trial soon after randomisation, and before the preintervention period during which schools were selected for inclusion in the trial. Given that pupil eligibility for the main trial was defined in advance as those who provided data at baseline *and* were in a class selected for inclusion in the trial, no pupils from this school are included in any summary statistics or analyses.

The characteristics of schools, teachers and students that took part in the trial are summarised in table 1. School classes within schools were selected to participate (n=8376 students). The study schools and students were broadly representative of the UK population (online supplemental F) and the SBMT and TAU groups were similar in terms of school and student characteristics.

**Table 1:** Baseline characteristics of schools and students by trial arm and overall

School (cluster) characteristics	SBMT arm	TAU arm	Total
	n=43	n=41	n=84
Region			
England, n (%)	38 (88)	36 (88)	74 (88)
Scotland, n (%)	2 (5)	1 (2)	3 (4)
Wales, n (%)	1 (2)	2 (5)	3 (4)
Northern Ireland, n (%)	2 (5)	2 (5)	4 (5)
School size—at least 1000 students, n (%)	20 (47)	22 (54)	42 (50)
Type of school			
Mixed, n (%)	36 (84)	37 (90)	73 (87)
Girls, n (%)	7 (16)	4 (10)	11 (13)
School quality rating			
Requires improvement, n (%)	6 (14)	5 (12)	11 (13)
Does not require improvement, n (%)	37 (86)	36 (88)	73 (87)
Deprivation			
Above median percentage eligible for free school meals, n (%)	15 (35)	15 (37)	30 (36)
Below median percentage eligible for free school meals, n (%)	28 (65)	26 (63)	54 (64)
Provision of Social Emotional Learning, mean (SD)	12 (2.5)	12 (2.6)	12 (2.6)

Student characteristics	SBMT arm	TAU arm	Total
	n=4232	n=4144	n=8376
Gender			
Female, n (%)	2350 (56.5)	2159 (53.1)	4509 (54.9)
Male, n (%)	1724 (41.5)	1823 (44.9)	3547 (43.2)
Other, n (%)	14 (0.3)	12 (0.3)	26 (0.3)
Prefer not to say, n (%)	69 (1.7)	69 (1.7)	138 (1.7)
Ethnicity—white, n (%)	3237 (78.1)	2965 (73.2)	6202 (75.7)
Age, mean (SD)	12.2 (0.6)	12.2 (0.6)	12.2 (0.6)
Year group			
Year 7, n (%)	2082 (49.2)	2142 (51.7)	4224 (50.4)
Year 8, n (%)	1878 (44.4)	1827 (44.1)	3705 (44.2)
Year 9, n (%)	79 (1.9)	64 (1.5)	143 (1.7)
Year S1, n (%)	193 (4.6)	111 (2.7)	304 (3.6)
Depression (CES-D), mean (SD)	13.6 (10.0)	13.3 (9.8)	13.5 (9.9)
Social-emotional and behavioural functioning (SDQ) total difficulties—self report, mean (SD)	11.8 (6.5)	11.7 (6.4)	11.8 (6.5)
Well-being (WEMWBS), mean (SD)	49.7 (9.7)	49.6 (9.7)	49.7 (9.7)

Data on baseline characteristics are provided for all 43 schools in the intervention arm and 41 of the 42 schools in the control arm (1 school withdrew soon after randomisation). No all boys schools were recruited.

Sample size ranges from 4145 to 4232 students in the intervention arm and 4048 to 4144 students in the control arm. In the intervention arm, 4157 students provided data on gender, 4145 students provided data on ethnicity, 4230 students provided data on CES-D, 4171 on SDQ and 4214 on WEMWBS. In the control arm, 4063 students provided data on gender, 4048 students provided data on ethnicity, 4140 students provided data on CES-D, 4081 on SDQ and 4119 on WEMWBS. Age and year group data are available for all students.

School year groups correspond across the home nations as follows: England 7, 8, 9 and 10; Northern Ireland Years 8, 9, 10 and 11; Scotland S1, S2 and S3.

CES-D, Center for Epidemiologic Studies for Depression Scale; SBMT, school-based mindfulness training; SDQ, Strengths and Difficulties Questionnaire; TAU, teaching as usual ; WEMWBS, Warwick-Edinburgh Mental Well-being Scale.

Of the students recruited to the trial, 86.6% (86.9% in the SBMT arm and 86.2% in the TAU arm) provided data on the co-primary outcomes at 1 year follow-up. The students retained at follow-up had more favourable mean (SD) scores at baseline than those lost to follow-up on risk for depression (13.1 (9.7) vs 15.5 (10.7)), social-emotional-behavioural functioning (11.5 (6·4) vs 13.5 (6.6)) and well-being (49.9 (9.6) vs 48.2 (10.2)). These mean scores at baseline were similar between the SBMT and TAU arms among those followed up (online supplemental table S2).

### Intervention dose, quality, fidelity and integration into school curriculum

The intervention was delivered as intended; students received an average of 9.0 (SD 2.1) out of a possible 10 SBMT sessions. On average, teachers were rated as delivering the intervention competently and adhered to 83% of the standardised curriculum. To assess the integration of the SBMT into the school curriculum, we surveyed all schools and crosschecked their responses with student attendance registers. SBMT was added to existing SEL provision for 53% of classes, substituted other SEL provision in 23% of classes, was partially additive/substitutive for 19% of classes and could not be established for the remainder. Given the complexity of curriculum management in secondary schools, provision mirrored the pragmatic reality of how schools offer SEL curricula.

### Student acceptability and engagement

Students’ ratings of the SBMT’s acceptability were heterogeneous (mean (SD): 4.7 (2.9) out of 10), with extreme reporting at either end of the scale and at the middle (see online supplemental G). Students’ engagement with the SBMT (home practice) was low at both postintervention (mean (SD): 1.16 (1.07) out of 5) and 1 year follow-up (mean (SD): 0.83 (0.93) out of 5).

### Student outcomes: effectiveness

Student outcomes are reported by trial arm status postintervention (table 2) and at 1 year follow-up (table 3).

**Table 2:** Main comparisons of student outcomes at postintervention follow-up

Secondary outcomes	SBMT arm (I)		TAU arm (C)		Unadjusted	Adjusted mean difference (I-C)/OR ratio (I/C)*			ICC†
	N	Mean (SD)/N (%)	N	Mean (SD)/N(%)	Mean diff. (I-C)/OR (I/C)*	Estimate	95% CI	P value	
Depression (CES-D)	3768	16.9 (11.8)	3793	16.4 (11.6)	0.4	0.2	−0.4 to 0.8	0.51	0.016
Social-emotional and behavioural functioning (SDQ)—self report									
Total difficulties	3752	13.4 (6.9)	3790	13.1 (6.8)	0.3	0.2	−0.1 to 0.6	0.15	0.018
Well-being (WEMWBS)	3775	47.8 (9.6)	3797	48.1 (9.3)	−0.2	−0.2	−0.7 to 0.3	0.44	0.015
Social-emotional and behavioural functioning (SDQ)—self report									
Emotional symptoms	3752	4.0 (2.8)	3790	3.8 (2.7)	0.2	0.1	−0.04 to 0.2	0.15	0.020
Conduct problems	3752	2.6 (2.0)	3790	2.6 (2.0)	0.007	−0.009	−0.1 to 0.1	0.86	0.015
Hyperactivity/inattention	3752	4.7 (2.6)	3790	4.5 (2.6)	0.1	0.2	0.04 to 0.3	0.01	0.015
Peer relationship problems	3752	2.2 (1.9)	3790	2.2 (1.9)	−0.001	0·0002	−0.1 to 0.1	1.00	0.012
Prosocial behaviour	3752	7.5 (2.0)	3790	7.4 (1.9)	0.008	−0.03	−0.1 to 0.1	0.54	0.021
Executive processing (BRIEF-2)—self-report	3115	85.7 (22.2)	3426	84.9 (21.9)	0.4	0.1	−1.5 to 1.7	0.90	0.014
Executive processing (BRIEF-2)—teacher-report	3083	80.2 (24.6)	2451	80.0 (24.5)	−0.3	0.2	−2.7 to 3.0	0.91	0.066
Drug use—self-report	3410	458 (13.4)	3621	524 (14.5)	1.0	0.9	0.8 to 1.1	0.56	0.015
Alcohol use—self-report	3428	1556 (45.4)	3630	1752 (48.3)	0.9	1.0	0.8 to 1.2	0.67	0.075
Anxiety (RCADS)—self-report									
Total score	3487	31.3 (21.6)	3688	29.1 (20.8)	1.6	1.3	−0.2 to 2.7	0.08	0.027
Social phobia	3492	10.9 (6.9)	3694	10.4 (6.6)	0.3	0.2	−0.2 to 0.6	0.40	0.041
Panic disorder	3487	6.5 (6.1)	3689	5.8 (5.9)	0.5	0.4	0.03 to 0.9	0.04	0.019
Separation anxiety	3491	3.4 (3.6)	3693	3.1 (3.5)	0.2	0.2	−0.05 to 0.4	0.12	0.017
Generalised anxiety	3499	6.2 (4.5)	3697	5.8 (4.3)	0.3	0.2	−0.1 to 0.5	0.18	0.027
Obsessive-compulsive	3496	4.4 (3.8)	3695	4.1 (3.7)	0.3	0.3	0.01 to 0.5	0.04	0.016
Social-emotional and behavioural functioning (SDQ)—teacher-report									
Total difficulties	3071	5.9 (6.0)	2451	5.6 (5.8)	0.3	0.3	−0.3 to 1.0	0.32	0.051
Emotional symptoms	3071	1.2 (1.9)	2451	1.0 (1.9)	0.1	0.2	−0.1 to 0.4	0.14	0.044
Conduct problems	3071	0.9 (1.7)	2451	0.8 (1.5)	0.1	0.1	−0.1 to 0.2	0.33	0.024
Hyperactivity/inattention	3071	2.5 (2.7)	2451	2.5 (2.7)	0.003	0.03	−0.2 to 0.3	0.82	0.044
Peer relationship problems	3071	1.3 (1.7)	2451	1.3 (1.7)	0.05	0.1	−0.1 to 0.3	0.48	0.035
Prosocial behaviour	3071	7.2 (2.6)	2451	7.2 (2.6)	−0.004	−0.05	−0.4 to 0.3	0.77	0.049
Self-harm—self-report	3389	365 (10.8)	3431	361 (10.5)	1.0	1.0	0.8 to 1.2	0.94	0.015
Suicide ideation—self-report	3251	745 (22.9)	3246	718 (22.1)	1.0	1.0	0.9 to 1.2	0.70	0.015
Mindfulness skills (CAMM)—self-report	3703	26.0 (8.3)	3769	26.7 (8.3)	−0.7	−0.6	−1.2 to −0.01	0.04	0.021
School ecology/climate (SCCS)									
School leadership and student involvement	3606	3.2 (0.8)	3749	3.2 (0.9)	0.006	0.009	−0.1 to 0.1	0.88	0.070
Respectful climate	3600	3.2 (0.8)	3746	3.3 (0.8)	−0.1	−0.04	−0.1 to 0.04	0.32	0.047
Peer climate	3598	3.0 (0.8)	3745	3.0 (0.8)	−0.02	−0.04	−0.1 to 0.05	0.41	0.061
Caring adults	3593	3.1 (0.9)	3744	3.2 (0.9)	−0.04	−0.02	−0.1 to 0.1	0.53	0.036

*Mean difference reported for quantitative outcomes and OR reported for binary outcomes.

†Intracluster (intraschool) correlation coefficients (ICCs) from crude (unadjusted) analyses.

BRIEF-2, Behaviour Rating Inventory of Executive Function, Second Edition; CAMM, Child and Adolescent Mindfulness Measure; CES-D, Center for Epidemiologic Studies for Depression Scale; I/C, intervention arm / control arm ; I-C, intervention arm - control arm; RCADS, Revised Child Anxiety and Depression Scale; SBMT, school-based mindfulness training; SCCS, School Climate and Connectedness Survey; SDQ, Strengths and Difficulties Questionnaire; TAU, teaching as usual; WEMWBS, Warwick-Edinburgh Mental Well-Being Scale.

**Table 3:** Main comparisons of student outcomes at 1 year follow-up

Outcome	Intervention arm (I)		Control arm (C)		Unadjusted	Adjusted mean difference (I-C)/OR (I/C)*			ICC†
	N	Mean (SD)/N (%)	N	Mean (SD)/N (%)	Mean diff. (I-C)/OR (I/C)*	Estimate	95% CI	P value	
**Co-primary outcomes**									
Depression (CES-D)	3672	17.1 (11.9)	3566	16.6 (11.9)	0.4	0.1	−0.6 to 0.7	0.86	0.018
Social-emotional and behavioural functioning (SDQ)—self report									
Total difficulties	3664	13.2 (6.8)	3561	12.9 (6.8)	0.2	0.2	−0.2 to 0.5	0.33	0.019
Well-being (WEMWBS)	3678	47.6 (9.8)	3566	47.6 (9.8)	0.1	0.2	−0.3 to 0.7	0.50	0.014
**Secondary outcomes**									
Social-emotional and behavioural functioning (SDQ)—self report									
Emotional symptoms	3664	4.0 (2.7)	3562	3.8 (2.7)	0.1	0.1	−0.1 to 0.2	0.25	0.019
Conduct problems	3664	2.4 (2.0)	3562	2.5 (2.0)	−0.04	−0.1	−0.2 to 0.1	0.30	0.014
Hyperactivity/inattention	3664	4.6 (2.6)	3562	4.5 (2.5)	0.2	0.2	0.04 to 0.3	0.01	0.015
Peer relationship problems	3664	2.2 (1.9)	3561	2.2 (1.9)	−0.01	−0.01	−0.1 to 0.1	0.78	0.016
Prosocial behaviour	3664	7.4 (2.0)	3562	7.4 (2.0)	0.03	−0.01	−0.1 to 0.1	0.81	0.025
Executive processing (BRIEF-2)—self-report	3288	84.3 (22.7)	3329	83.6 (22.4)	0.5	0.2	−1.4 to 1.7	0.84	0.019
Executive processing (BRIEF-2)—teacher report	2489	77.8 (22.8)	1990	78.7 (24.3)	−0.3	0.02	−2.8 to 2.9	0.99	0.063
Drug use—self-report	3401	587 (17.3)	3429	635 (18.5)	0.9	0.9	0.8 to 1.1	0.38	0.016
Alcohol use—self-report	3436	1703 (49.6)	3451	1729 (50.1)	1.0	1.0	0.8 to 1.3	0.77	0.078
Anxiety (RCADS)—self-report									
Total score	3504	30.0 (21.5)	3483	28.8 (21.6)	1.0	0.4	−1.0 to 1.9	0.56	0.026
Social phobia	3510	10.6 (6.9)	3488	10.3 (6.8)	0.2	−0.01	−0.4 to 0.4	0.96	0.033
Panic disorder	3504	6.2 (6.1)	3485	5.8 (6.1)	0.4	0.2	−0.2 to 0.6	0.30	0.022
Separation anxiety	3508	3.2 (3.5)	3488	3.1 (3.6)	0.2	0.1	−0.1 to 0.3	0.45	0.016
Generalised anxiety	3512	5.9 (4.4)	3490	5.7 (4.4)	0.1	0.03	−0.3 to 0.3	0.86	0.025
Obsessive-compulsive	3512	4.1 (3.7)	3489	3.9 (3.8)	0.2	0.1	−0.1 to 0.4	0.39	0.017
Social-emotional and behavioural functioning (SDQ)—teacher-report									
Total difficulties	2496	5.3 (5.7)	1981	5.1 (5.8)	0.5	0.6	−0.2 to 1.3	0.14	0.065
Emotional symptoms	2496	1.1 (1.9)	1981	1.0 (1.8)	0.3	0.3	0.1 to 0.5	0.01	0.053
Conduct problems	2496	0.7 (1.5)	1981	0.8 (1.5)	0.05	0.1	−0.1 to 0.2	0.48	0.035
Hyperactivity/inattention	2496	2.2 (2.6)	1981	2.2 (2.6)	0.1	0.1	−0.2 to 0.4	0.59	0.049
Peer relationship problems	2496	1.2 (1.7)	1981	1.2 (1.7)	0.1	0.1	−0.1 to 0.3	0.31	0.046
Prosocial behaviour	2496	7.6 (2.5)	1981	7.5 (2.6)	0.1	0.1	−0.3 to 0.4	0.76	0.086
Self-harm—self-report	3364	389 (11.6)	3234	385 (11.9)	1.0	0.9	0.8 to 1.1	0.53	0.012
Suicide ideation—self-report	3224	779 (24.2)	3098	709 (22.9)	1.1	1.1	0.9 to 1.2	0.45	0.013
Mindfulness skills (CAMM)—self-report	3625	26.4 (8.5)	3546	26.7 (8.7)	−0.3	−0.1	−0.6 to 0.5	0.78	0.019
School ecology/climate (SCCS)									
School leadership and student involvement	3587	3.1 (0.9)	3530	3.1 (0.9)	−0.01	−0.01	−0.1 to 0.1	0.85	0.072
Respectful climate	3582	3.2 (0.8)	3527	3.2 (0.8)	−0.01	−0.01	−0.1 to 0.1	0.89	0.038
Peer climate	3581	3.0 (0.7)	3523	3.0 (0.7)	−0.004	−0.01	−0.1 to 0.1	0.74	0.060
Caring adults	3576	3.1 (0.9)	3518	3.2 (0.9)	−0.04	−0.03	−0.1 to 0.04	0.48	0.031

*Mean difference reported for quantitative outcomes and OR reported for binary outcomes.

†Intracluster (intraschool) correlation coefficients (ICCs) from crude (unadjusted) analyses.

BRIEF-2, Behaviour Rating Inventory of Executive Function, Second Edition; CAMM, Child and Adolescent Mindfulness Measure; CES-D, Center for Epidemiologic Studies for Depression Scale; RCADS, Revised Child Anxiety and Depression Scale; SCCS, School Climate and Connectedness Survey; SDQ, Strengths and Difficulties Questionnaire; WEMWBS, Warwick-Edinburgh Mental Well-Being Scale.

At 1 year follow-up (the primary end point) there was no evidence of a difference for any of the co-primary outcomes (table 3). Furthermore, when converting the estimated differences to the effect size scale (calculated as the adjusted mean difference divided by the pooled SD of the outcome measure) we can rule out the possibility of important effects based on the 95% CIs. The effect sizes (95% CI) are: 0.005 (−0.05 to 0.06) for risk for depression (CES-D); 0.02 (−0.02 to 0.07) for social-emotional-behavioural functioning (SDQ); and 0.02 (−0.03 to 0.07) for well-being (WEMWBS). Tests of interaction revealed little evidence that the intervention effect on the three co-primaries differs between Cohorts 1 and 2 (interaction p values: 0.61, 0.89 and 0.86, respectively).

Only for five of 28 secondary outcomes was there some evidence of a difference between the trial arms. Intervention arm students had *higher* self-reported hyperactivity/inattention on the SDQ subscale at both postintervention and 1 year follow-up, and *higher* panic disorder and obsessive-compulsive scores on the RCADS measure at postintervention, *lower* levels of mindfulness skills on the CAMM postintervention only plus *higher* teacher-reported emotional symptoms on the SDQ at 1 year follow-up only, suggesting that they are doing *worse*, although marginally, on these outcomes than the control arm. The conclusions of the sensitivity analyses of the modified intention-to-treat population were robust to those of the primary analyses (see online supplemental table S4). The pattern of findings was also consistent postintervention; there were no statistically significant differences between the SBMT and TAU arms on any primary or secondary outcome (online supplemental table S3).

### Student outcomes: cost-effectiveness

**Figure 2:**
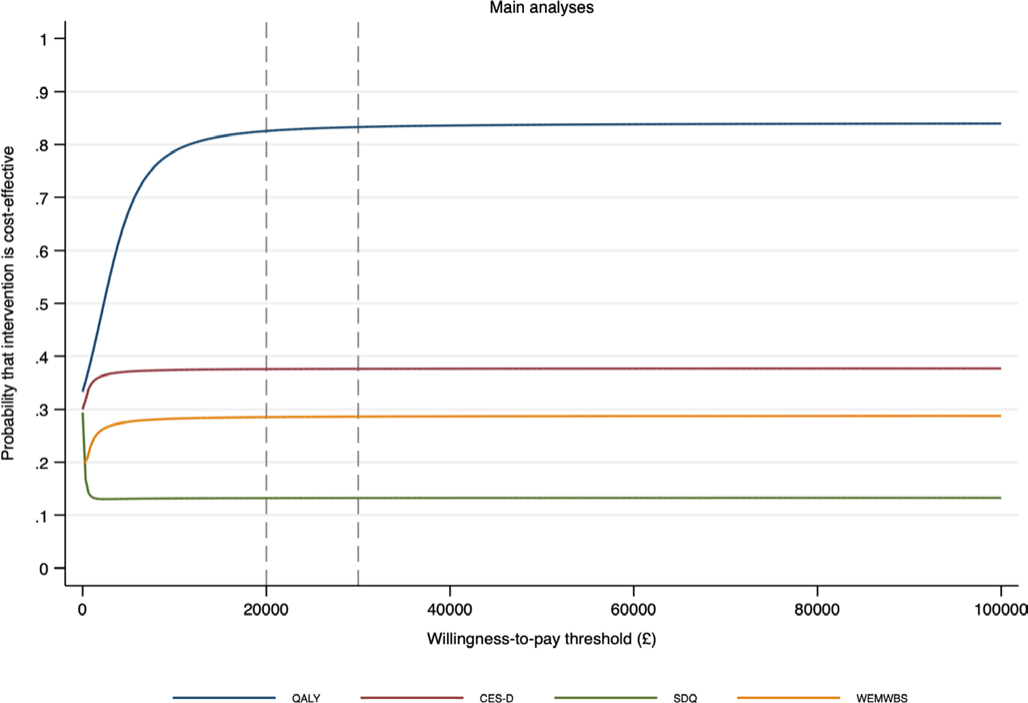
Cost-effectiveness acceptability curves for the primary economic analyses using quality adjusted life years (QALYs).

The mean cost of the SBMT intervention was £70.73 per student. Data used in the economic analyses, including costs, CHU9D utilities and QALYs, and the co-primary outcome measures are reported in table 4. Total mean costs and QALYs were higher in the SBMT arm (mean costs £1333.57, SD=£2389.40; mean QALYs 0.871, SD=0.130) than the TAU arm (mean costs £1290.79, SD=£1379.13; mean QALYs 0.847, SD=0.131). However, differences between trial arms in both costs and QALYs were small in adjusted analyses (adjusted mean difference in cost £6.84, 95% CI −£128.04 to £141.72; adjusted mean difference in QALYs 0.012, 95% CI −0.015 to 0.038). Primary cost-utility analysis using QALYs as the outcome suggests that SBMT has a high probability (83%; figure 2) of being cost-effective for all willingness-to-pay thresholds used by the National Institute for Health and Care Excellence (£20 000 to £30 000 per QALY). All sensitivity analyses suggest that SBMT is cost-effective (probability >50%) under these same thresholds. Secondary economic analyses, using the co-primary outcomes as the measures of effect suggest that SBMT has a low probability of being cost-effective (<40%) for all willingness-to-pay thresholds. Full details of all economic analyses are reported in the online supplemental H.

**Table 4:** Mean costs in UK pounds sterling (£) per student and health-related quality-of-life outcomes at 1 year follow-up

	Intervention arm (I)		Control arm (C)		Unadjusted mean difference (I-C)	Adjusted mean difference (I-C)		
	N	Mean (SD)	N	Mean (SD)		Estimate	95% CI	P value
Costs (£)								
Preintervention	4080	360.28 (1,242.25)	3995	378.90 (1,444.81)	−18.62	−18.81	−84.96 to 47.33	0.57
Intervention	3424	70.73 (21.66)	3370	0.00 (0.00)	70.73	71.61	64.84 to 78.38	<0·0001
Hospital	3424	601.55 (1,569.74)	3370	636.51 (1,660.74)	−35.73	−36.70	−120.64 to 47.24	0.39
Community health and social care	3424	377.92 (1,014.23)	3370	636.51 (1,660.74)	−6.14	−21.97	−73.21 to 29.27	0.40
Medication	3424	17.18 (76.98)	3370	18.81 (83.44)	−2.74	−2.08	−6.94 to 2.78	0.40
Accommodation	3424	23.54 (458.70)	3370	18.03 (426.27)	5.51	5.30	−14.96 to 25.57	0.61
Teaching support	3424	242.59 (810.92)	3370	232.54 (755.87)	3.72	−6.33	−53.35 to 40.69	0.79
Total	3424	1,333.57 (2,389.42)	3370	1,290.79 (1,379.13)	34.90	6.84	−128.04 to 141.72	0.92
Health-related quality of life (CHU9D)								
Utility score preintervention	4029	0.838 (0.118)	3953	0.837 (0.116)				
Utility score postintervention	3736	0.825 (0.127)	3779	0.828 (0.124)				
Utility score at 1 year follow-up	3651	0.824 (0.129)	3551	0.823 (0.129)				
Total QALYs	3313	0.871 (0.130)	3287	0.847 (0.131)	0.017	0.012	−0.015 to 0.038	0.39

CHU9D, Child Health Utility 9D; QALY, quality-adjusted life year.

### Serious adverse events

Two serious adverse events were recorded, both in teachers (terminal illness diagnosis and death from natural causes), both in the SBMT arm. Review by the independent data monitoring and ethics committee concluded that neither was attributable to the SBMT.

## Discussion

This cluster randomised controlled trial is the largest evaluation of the effectiveness and cost-effectiveness of a universal SBMT programme in early adolescence on mental health and well-being outcomes. There was no evidence for the superior effectiveness of SBMT compared with usual social-emotional learning provision in terms of any of the co-primary or secondary outcomes. Economic analysis suggests SBMT has a higher probability of being cost-effective than standard teaching in terms of QALYs, a finding which was robust to sensitivity analyses, and at the £20 000 willingness-to-pay thresholds used by the National Institute for Health and Care Excellence. However, these results were generated by small differences in costs and QALYs and there was no evidence to support the cost-effectiveness of SBMT when considering primary outcomes. There was some evidence of SBMT being associated with worse outcomes than TAU on five secondary measures, a significant finding suggesting the need to pause and take stock before further implementation of universal SBMT.

Prior to this study, a systematic review and meta-analysis suggested SBMT showed promise in terms of effectiveness on mental health outcomes, but pointed to the need for larger, more rigorous randomised trials with longer follow-ups.^6^ Since the present study started, there have been at least 40 further studies of SBMT.

Theory and research highlight that several key issues need to be considered for universal SEL interventions to be acceptable, effective and sustainable.^26^ At the societal level, external influences on young people’s well-being, such as deprivation and inequality are major determinants of mental health.^3^ At the school-level implementation facilitators and barriers are key.^14^ For example, a filter operated between those schools approached to take part in the research and those that responded and were deemed eligible (85/5663). At the programme level fidelity, credibility, engagement and cultural sensitivity need consideration.^27^ At the student level variation mental health status and age/developmental stage, and trajectories of change need consideration.^4^ These issues suggest a number of possible reasons that universal SBMT was not more effective than TAU in this trial. Given that mindfulness as taught in this SBMT curriculum involves meta-cognitive awareness, it may be that it is indicated only for older adolescents. The SBMT curriculum we used may simply not be intensive enough to create changes in the hypothesised mechanisms of enhancing attention and self-regulation skills, especially as we found that young people have very mixed views of the acceptability of SBMT, and largely did not practise the skills at home. A mindfulness intervention in this age group might better target points of clear need (eg, exam stress, sleep, hygiene), explicitly address motivation to practice these skills and use modalities that are more accessible (eg, online). Moreover, the differences suggesting worse outcomes for SBMT versus TAU in terms of hyperactivity/inattention and emotional problems could be attributable to several factors. It may be that a universal intervention like SBMT has different effects on different subgroups, with some benefiting and others worsening.^4^ Mindfulness training involves asking people to become more aware of thoughts and feelings, including unpleasant ones. It is possible in this setting, with this curriculum and these teachers, this can exacerbate difficulties, at least for some students. It is possible that other SEL curricula might be even more effective than the curriculum we used or that the curriculum we used needs to be enhanced, especially with respect to acceptability, engagement and tools to work with distressing socioemotional behavioural issues. Co-designing such curricula with young people might enhance acceptability. This SBMT curriculum was delivered by school teachers with the rationale that they already know the students and have good general teaching skills. However, it may be that school teachers are not ideally placed to offer SBMT because they have a particular school teacher-student relationship that mitigates against SEL instruction. In addition, the skills required to teach SBMT require an approach that is arguably pedagogically orthogonal to mainstream academic teaching and might not readily be acquired alongside the many other demands on school teachers. The rates of acceptability and engagement also point to MT that *requires participation* being contraindicated. That is to say, we should not ask students to do something they don’t like or engage with, when there are no clear benefits and which for some might, at least for students, exacerbate their mental health difficulties. Finally, given that schools are complex environments, with many competing priorities, it is essential that we use extant evidence of which SEL curricula are most likely to improve the mental health and well-being of children in schools. Moreover, that we support schools using the implementation science evidence to integrate these curricula in a sustainable way. Finally, that education policy likewise is informed by this evidence.

This study has a number of noteworthy strengths. It was fully powered and the sample was representative of UK schools and young people. The SBMT was delivered with satisfactory fidelity. We had good retention at the primary end point (87%). Schools incorporated SBMT pragmatically, some adding it and others substituting it for other provisions, making this a test of effectiveness rather than efficacy. The external validity was maximised by the generalisability of the sample, relatively long follow-up (1 year), and attention to real world implementation and sustainability.

The study also had a number of limitations. We used adolescent self-report extensively, which, while appropriate for some measures (eg, co-primary outcomes), may have been less so for others (eg, collection of resource use data through adolescent self-report for the economic evaluation may have reduced the validity of these data). However, we included teacher-reported versions of several secondary outcomes (eg, social-emotional-behavioural functioning) to mitigate this. Students and teachers were necessarily not blind to treatment allocation. We only included schools with adequate SEL provision, and it is possible that if we had been able to compare SBMT with a setting in which there was no SEL provision we may have found an effect. Finally, our original intention was to include a longer 2 years follow-up, but the COVID-19 pandemic prevented this.^10^ It is possible that effects of SBMT could take longer to emerge, especially if an intervention could engage young people more fully in behaviour change that meaningfully enhances resilience. Depression typically emerges in adolescence/early adulthood, and understanding and changing the trajectories of vulnerability and resilience in this window are key topics for future research.

## Conclusions

In a fully powered, rigorous, cluster randomised controlled trial we found no support for our hypothesis that SBMT is superior in terms of mental health and well-being compared with usual provision over 1 year of follow-up in young people in secondary schools. While SBMT had marginally superior cost-effectiveness based on QALYs, there was no evidence of a cost-effective advantage using the co-primary outcome measures. This has significant implications for policy and for schools where the enthusiasm for SBMT has led to implementation ahead of the evidence. We need to move to asking, ‘What works, for whom, and how?’. Our scoping review sets out a conceptual model with testable hypotheses.^7^ In a parallel paper we report MYRIAD Trial effects on teacher-level (mental health and functioning) and school-level (eg, climate) outcomes.^28^ In a further paper, we explore potential moderators (eg, student age), mechanisms (eg, participants’ engagement) and implementation facilitators (eg, teacher competency).^29^

## Data Availability

Data are available upon reasonable request. The baseline data and codebook from the MYRIAD Trial are available from Prof Kuyken (willem.kuyken@psych.ox.ac.uk) upon request (release of data is subject to an approved proposal and a signed data access agreement).
