# Supplementary material for: Effectiveness and cost-effectiveness of universal school-based mindfulness training compared with normal school provision in reducing risk of mental health problems and promoting well-being in adolescence: the MYRIAD cluster randomised controlled trial
Source: Evid Based Ment Health. 2022 Jul 7;25(3):99–109. doi: 10.1136/ebmental-2021-300396 (PMC9340028; doi:10.1136/ebmental-2021-300396)
Supplement: Supplementary data [file ebmental-2021-300396supp001.pdf]

Effectiveness and cost-effectiveness of universal school-based mindfulness training compared with normal school provision in reducing risk of mental health problems and promoting well-being in adolescence: the MYRIAD cluster randomised controlled trial

Supplements

Supplement A: SBMT program (.b) and Teaching as Usual (TAU). Page 2

Supplement B: Pupil, teacher, school measures. Page 5

Supplement C: Cost. Page 13

Supplementary Table S1: Unit cost and sources. Page 14

Supplement D: Further details of economic methods. Page 16

Supplement E: Further details on the CONSORT diagram. Page 17

Supplement F: Representativeness of study schools and pupils. Page 18

Supplement G: Frequencies of pupil's ratings of the SBMT's acceptability. Page 19

Supplementary Table S2: Selected study/trial pupil baseline characteristics by 1 year follow-up status. Page 20

Supplementary Table S3: Main comparisons of pupil outcomes at post-intervention follow-up: modified intent-to-treat (complete case) analysis. Page 21

Supplementary Table S4: Main comparisons of pupil outcomes at 1 year follow-up: modified intent-to-treat (complete case) analysis. Page 23

Supplement H: Details of all economic analyses. Page 25

References. Page 35

Kuyken W, *et al. Evid Based Ment Health* 2022; 25:99–109. doi: 10.1136/ebmental-2021-300396

Supplement A

SBMT programme (.b)

The SBMT programme's primary aim was to teach mindfulness skills that support young people's resilience. Mindfulness is a natural and trainable capacity to bring awareness to both inner (e.g., thoughts, feelings, body sensations) and outer experience (e.g., stressors, relationships) with qualities of curiosity and kindness. The SBMT programme we used was developed and adapted from mindfulness-based cognitive therapy (Segal et al., 2013) over more than 5 years by three classroom teachers (Richard Burnett, Chris Cullen and Chris O'Neill) who are also experienced mindfulness practitioners. Their aim was to make it acceptable to young people across the full spectrum of functioning from mental health problems to flourishing, enabling all young people to use mindfulness skills to manage emotions, academic study, sport, sleep and relationships. This has included ensuring that the programme can be taught in mainstream schools, how best to engage hard-to-reach children and how to manage challenging classroom behaviour.

The SBMT programme comprised several elements, delivered through the school curriculum, over several years, supported by teacher training. The bulk of the SBMT programme was taught to students in a set of 10 (30-50 minutes each) structured lessons (within the trial, taught in English years 8 and/or 9). The SBMT programme was normally be delivered in the spring terms (January through April), with support to continue use of mindfulness skills into the summer term. In the following school years, there are booster lessons intended to continue and support pupils' further learning and ongoing mindfulness practice (e.g. lunchtime clubs or drop-in sessions). This follow-on training in subsequent school years aims to sustain, deepen and begin to apply students' learning; for example, to managing tests and examinations and to embed mindfulness in the school ecology/climate.

The SBMT programme includes a combination of psycho-education and practical skills involved in training the mind, learned in an experiential way, through short mindfulness practices which focus on the breath, body and immediate experience. There is also classroom discussion of the application of new skills in everyday life. Its design aligns with principles identified as important for effectiveness in several reviews of schools-based programmes that promote student mental health and wellbeing and teach social and emotional competence. These principles include: explicitly teaching skills and attitudes; tailoring components and approaches to the needs of young people; using a range of age-appropriate, interactive, experiential and lively teaching methods; providing age- appropriate resources; for example, in this context resources that bring mindfulness to life (including a course booklet, a set of mindfulness exercises provided online and mindfulness practices that are introduced through animations and available as digital downloads); intensive, focused teacher education to build teachers' self-efficacy and wellbeing; and programme implementation which pays close attention to clarity and fidelity, in this case supported by a manual and indicative script (Mindfulness in Schools Project, 2013). Building on data that greater practice is associated with better outcomes (Huppert & Johnson, 2010; Kuyken et al., 2013), the SBMT programme includes strategies to support teachers in keeping mindfulness integral to the culture of their year group/the school as a whole. Examples of good practice in this area could include teacher catch-up days/support events, suggested schedules for progressive, regular

Kuyken W, *et al. Evid Based Ment Health* 2022; 25:99–109. doi: 10.1136/ebmental-2021-300396

mindfulness input throughout year groups, suggested smartphone apps and using parts of the SBMT programme in core curriculum subjects.

Our approach to implementing the SBMT was informed by theory and implementation science (Tudor et al., under review), and was designed to be fully integrated into the school curriculum, over several years. Because implementation affects both reach and outcomes, all schools were supported with implementation guidance to increase the likelihood that it was introduced into the schools in ways that maintain its integrity and are sustainable. Implementation started with engaging the school leadership team, and then identifying a potential pool of teachers from within the school who could be trained and timetabled to deliver it to the pupils. The selected teachers then went through a training programme (see below).

All participating schools randomised to SBMT agreed to deliver the SBMT programme to a minimum of three classes within years 8 and/or 9 or equivalent year groups across the nations (pupils aged 12-14), but were also encouraged to consider how they might introduce mindfulness into the curriculum more broadly, for the potential benefit of other school pupils and the wider school climate.

The SBMT teacher training involved first participating in an 8-week personal mindfulness-based cognitive therapy for life (MBCT-L) programme. MBCT-L was developed as a mindfulness training for the general population that supports resilience and well-being. The programme comprises eight two-hour sessions per week, with an all-day mindfulness session supported by a course handbook and online mindfulness practices (Kuyken et al., manuscript in preparation). Participants are encouraged to develop a daily mindfulness practice, both during the MT and to sustain this in an ongoing way afterwards. From the pool of teachers undergoing personal mindfulness training, schools selected the sample of teachers to go forward with the SBMT. Senior leadership teams in schools based selection on whether teachers would be willing and available to attend the further training and could be timetabled to teach the SBMT to participating study classes. Teachers who were selected to progress then attended a 4-day training workshop to learn how to deliver the SBMT curriculum to students. Following this 4-day training, participating teachers taught at least one complete SBMT curriculum to students, with support from an experienced mentor, before going on to teach the study students.

Within participating schools, as many teachers as possible were invited to attend the personal mindfulness training, to give schools the best opportunity to timetable the required number of teachers to teach the SBMT curriculum to study classes. Further embedding SBMT into the school included opening the training up to staff beyond the nominated teachers, helping schools integrate mindfulness into their school improvement plan by providing document templates, making mindfulness practice part of teacher catch-up days, professional development days, suggested schedules for progressive, regular mindfulness input throughout year groups (e.g., during assemblies), suggested free smartphone apps for both students and teachers and using mindfulness skills throughout the school curriculum.

We have reported separately on the acceptability, effectiveness and cost-effectiveness of this teacher training (Crane et al., 2020) as well as the relative merits of less and more intensive training for school teachers in terms of acceptability, effectiveness, and mechanisms (Montero-Marin et al., in press). Based on the findings from this work the teachers received

the more intensive mindfulness curriculum as well as ongoing support to implement and deliver the SBMT curriculum to pupils.

Teaching as usual

The trial aimed to establish if SBMT, when integrated into social-emotional teaching in secondary schools, adds value over and above current good practice. Recent UK Department of Education reports suggest that 60% of secondary schools offer Personal, Social, Health and Economic Education (PSHE) lessons that are 'good or better' and that this provision occurs across ages 11–16 years (Key Stages 3 and 4) through a variety of methods including regular scheduled lessons, drop-down days, within other subjects, and in tutor/form time. (Department of Education, 2010). Determining whether schools have good PSHE provision is challenging. In cohort 1, schools were eligible for inclusion if their provision of PSHE (or equivalent) met four criteria: (1) the presence of discrete, regular, named teaching time for PSHE, (2) a named PSHE lead, (3) a written PSHE policy and (4) a named member of the senior leadership team responsible for PSHE. However, for cohort 2, the 'written SEL policy' criterion was modified to "documentation denoting clear strategic planning of SEL within the school." Experience in cohort 1 indicated that schools do not always use the term 'SEL policy' to denote strategic planning of SEL. Moreover, in some cases, there are schools that have an extensive, well-established and well-documented SEL curriculum, indicative of a clear structure and strategy around SEL, but do not have this formalised as a school policy. TAU schools agreed not to provide the MT programme (or other curricula that include MT) until study completion.

While SEL provision was not uniform in the TAU arm, content is intended to prepare students with the knowledge, skills and attributes they need to manage their lives. It typically covers relationships, sex education, and physical and mental health education.

Supplement B

Pupil Measures

Demographics

Pupil demographics, including gender (male, female), ethnicity (White, Arab/Arab British, Black/African/Caribbean/Black British, Mixed/Multiple Ethnic Groups, Other Ethnic Group), were gathered via pupil self-report at baseline. Year group (year 7, year 8, year 9, year S1), and dates of birth were reported by the school, and the research group calculated the corresponding age.

Co-primary outcomes

Center for Epidemiologic Studies for Depression Scale (CES-D; Radloff, 1991).

The "Center for Epidemiologic Studies for Depression Scale" (CES-D; Radloff, 1991) is a 20-item self-report questionnaire that assesses depressive symptoms in the past week (e.g., "I felt depressed"). It has been validated for use in adolescents (Radloff, 1991). Each item is rated on a rating-scale from 0 ("rarely or none of the time"), to 3 ("most or all of the time"), yielding a total score between 0 and 60, with higher scores indicating greater risk for depression. The following two cut-off points have been proposed: a) a lower cut-point of 16, to identify pupils at risk of depression (Rushton, Forcier & Schectman, 2002), and b) a higher cut-point of 28 to identify pupils with symptoms likely to meet diagnostic criteria for major depressive disorder (Radloff, 1991). The Cronbach's alpha value of the CES-D in our study at baseline were $\alpha = 0{\cdot}88$, being of $\alpha = 0{\cdot}91$ at pre-intervention, of $\alpha = 0{\cdot}92$ at post-intervention, and of $\alpha = 0{\cdot}92$ at 1-year follow up.

Strengths and Difficulties Questionnaire (SDQ, Goodman, 2001)

The "Strengths and Difficulties Questionnaire" (SDQ; Goodman, 2001) is a 25-item questionnaire that assesses social, emotional, and behavioural strengths and difficulties over the previous 6 months (e.g., "I am constantly fidgeting or squirming"). Each item is rated on a rating-scale from 0 ("not true"), to 2 ("certainly true"). The SDQ's five sub-scales assess emotional symptoms, conduct problems, hyperactivity/inattention, peer problems, and pro-social behaviour. The total difficulties score measures social/emotional/behavioural functioning (range 0-40), and is derived by summing the first four subscales, where higher scores indicate greater dysfunctional levels. Higher scores on the pro-social subscale, in contrast, indicate better social functioning (although subscale scores have not been used in the present study). SDQ total scores can be interpreted using either the 4-band categorisation; close to average (0-14), slightly raised (15-17), high (18-19) and very high (20-24); or the 3-band categorisation, normal (0-15), borderline (16-19), abnormal (20-40) (Goodman et al.,

2000). Pupil and teacher reports of the SDQ were used in the present study, with internal consistency values for pupils at baseline of α = 0·83, being of α = 0·84 at pre-intervention, of α = 0·85 at post-intervention, and of α = 0·85 at 1-year follow up; and internal consistency values for teachers of α = 0·89 at pre-intervention, of α = 0·89 at post-intervention, and of α = 0·86 at 1-year follow up.

Warwick-Edinburgh Mental Well-being Scale (WEMWBS; Tennant et al., 2007)

The "Warwick-Edinburgh Mental Well-being Scale" (WEMWBS; Tennant et al., 2007) is a 14-item measure assessing both feeling and functioning aspects of mental well-being over the last two weeks (e.g., "I've been feeling useful"). Items are scored on a rating-scale from 1, "none of the time" to 5, "all of the time", yielding a total score that ranges between 14 and 70. Items are worded positively and therefore higher scores indicate greater levels of mental well-being. The WEMWBS measure has been validated for its specific use in adolescents (Clark et al., 2011). There are not established cut-offs for the WEMWBS, although using M±1SD has been suggested. The internal consistency value of the WEMWBS in our study at baseline was α = 0·88, being of α = 0·87 at pre-intervention, of α = 0·89 at post-intervention, and of α = 0·91 at 1-year follow up.

Secondary Outcomes

Students' executive processing peer relationships drug and alcohol use (measure designed for study); anxiety (anxiety subscales from the Revised Child Anxiety and Depression Scale; RCADS (Weiss & Chorpita, 2011)); social-emotional and behavioural functioning (SDQ, teacher version; (Goodman, 2001)); self-harm and suicidal ideation (measures devised for study), and; mindfulness skills (Child-Adolescent Mindfulness Measure, CAMM (de Bruin et al., 2013)).

Behaviour Rating Inventory of Executive Function, Second Edition (BRIEF2; Gioia et al., 2015)

The "Behaviour Rating Inventory of Executive Function, Second Edition" (BRIEF2, Gioia et al., 2015) is a 55-item self-report measure designed to assess self-perception of everyday behaviours associated with executive function in older children and adolescents (aged 11-18), e.g., "I have trouble sitting still". The BRIEF-2 assesses executive function across the past 6 months and takes into account 7 domains: inhibit; self-monitor; shift; emotional control; task completion; working memory; and plan/organize. Items are rated as follows: 1 = "never", 2 = "sometimes", 3 = "often". Total scores are calculated by summing the sub-scores, with higher scores suggesting higher levels of executive dysfunction. The three items of the infrequency scale ("I forget my name", "I have trouble counting to three", "I cannot find the front door of my home") are only used as indicators of validity and are not included in the calculation of raw scale scores, so that the total score ranges between 52 and 156, and therefore higher scores meaning worse executive functioning. The pupil-report of this inventory was used in the present study. The internal consistency (Cronbach's alpha value) of the BRIEF-2 at pre-intervention was α = 0.97, α = 0.97 at post-intervention, and α = 0.97 at 1 year follow up.

Drug and alcohol use (risk measure)

Kuyken W, *et al. Evid Based Ment Health* 2022; 25:99–109. doi: 10.1136/ebmental-2021-300396

The Risk Measure contains seven questions assessing risk behaviours split into three subscales: smoking, drinking, and cannabis and other substances (https://www.thereachstudy.com/uploads/7/3/2/1/73211845/reach_protocol.pdf and http://www.espad.org). The questions gauge frequency of cigarette, alcohol, and cannabis use and include binary “yes/no” questions, e.g., “Have you used cannabis ever?”, as well as frequency scales, e.g., “Which of the following apply to you. I drink alcohol:” ranging from 0, “never”, to 6, “every day or almost every day”. The “Cannabis and Other Substances” subscale asks whether participants have tried a list of 11 substances/substance categories, e.g., “amphetamines/methamphetamine (e.g., speed, crystal meth)” with binary “yes/no” response options. It includes one “dummy” substance, “Decopan”; if a participant responded ‘yes’ to this item, all drug positive responses to this section of the risk measure were excluded from analysis. In addition, if a participant responded ‘yes’ to painkillers and no other drugs in this section, the positive painkiller response was excluded from analysis.

The Revised Children’s Anxiety and Depression Scale (RCADS; Weiss & Chorpita, 2011)

The “Revised Children’s Anxiety and Depression Scale” (RCADS; Weiss & Chorpita, 2011) is a youth self-report questionnaire that measures separation anxiety disorder (SAD), social phobia (SP), generalized anxiety disorder (GAD), panic disorder (PD), obsessive compulsive disorder (OCD), and major depressive disorder (MDD). Adapted for the MYRIAD project, the questions that make up the depression subscale were removed, to leave a 37-item questionnaire measuring anxiety, asking ‘how often’ each item happens (e.g., “I worry when I think I have done poorly at something”). Measured using a 4 point Likert-type scale (0 = Never, 1 = Sometimes, 2 = Often, and 3 = Always), the questionnaires subscales are scored by summing the items. A total Anxiety Scale can also be calculated by summing the 5 subscales. The internal consistency (Cronbach’s alpha value) of the RCADS total score at pre-intervention was $\alpha = 0.96$, being of $\alpha = 0.96$ at post-intervention and of $\alpha = 0.96$ at 1-year follow up.

Suicide and Self-harm

Suicide ideation and self-harm were measured using three self-report items (Madge et al., 2008). Participants were asked to consider their thoughts and behaviour since the last MYRIAD school visit. They could respond "yes", "no" or "prefer not to say" to the following statements: "Have you thought that life was not worth living, or that you would be better off dead?"; "Have you thought seriously about trying to harm yourself in some way (for example by cutting yourself or taking an overdose of pills or other medication)?", and "Have you actually, deliberately harmed yourself in some way (for example by cutting yourself or taking an overdose of pills or other medication)?".

The Child and Adolescent Mindfulness Measure (CAMM; Greco et al., 2010)

The “Child and Adolescent Mindfulness Measure” (CAMM; Greco et al., 2010) is a self-report mindfulness skills scale designed specifically for use with children and adolescents. It consists of 10-items, which measure awareness of the present moment as well as non-judgemental and non-avoidant responses to thoughts and feelings (e.g., “I keep myself busy so I don’t notice my thoughts or feelings”). Participants are asked how often each sentence is true, and responses are given using a 5-point Likert-type rating-scale ranging from 0, “Never True”, to 4, “Always True”. Each item is reverse scored and summed, producing a total score of 0-40, with higher scores corresponding to higher levels of mindfulness. The CAMM has

been validated for use in non-clinical samples of adolescents (de Bruin et al., 2013; Kuby et al., 2015), and has adequate psychometric properties (Greco et al., 2010). The internal consistency (Cronbach's alpha value) of this measure of mindfulness skills at pre-intervention was α = 0.84, α = 0.86 at post-intervention and o α = 0.88 at follow up.

School Climate and Connectedness Survey (SCCS; Association of Alaska School Boards, 2015)

The pupil version of the "School Climate and Connectedness Survey" (SCCS) measures aspects of school climate (social and environmental factors that contribute to the subjective experience of a school), and connectedness (perceptions and feelings about the people at school) for students, asking them to consider the way the school is 'most of the time' (e.g., "Teachers here are nice people"). There are 63 questions (9 subscales) on the original SCCS scale, all with 5-point Likert-type responses (e.g., from 1, "strongly agree", to 5, "strongly disagree"). For the current study, 21 questions (4 subscales) from the original SCCS questionnaire were employed: "School Leadership and Student Involvement Scale", "Respectful Climate", "Peer Climate Scale" and "Caring Adults Scale". Total scores were calculated by summing the corresponding subscale scores, with higher scores reflecting a more positive school climate and connectedness. The internal consistency of this measure of school climate at pre-intervention was α = 0.91, being of α = 0.91 at post-intervention and of α = 0.91 at 1-year follow up.

The Child Health Utility 9D (CHU9D; Stevens, 2011)

The "Child Health Utility 9D" (CHU9D) is a preference-based measure of health-related quality of life in young people. This is suitable for the calculation of Quality Adjusted Life Years (QALYs) and has been shown to be valid and responsive to change in adolescent populations (Furber & Segal, 2015). It includes nine health dimensions: worried, sad, pain, tired, annoyed, schoolwork, sleep, daily routine, and ability to join in with activities (not all of these dimensions are 'health dimensions'). Each dimension has five levels increasing with severity (e.g., from 1 = "I don't feel worried today" to 5 = "I feel very worried today"). Scores were produced using the UK SPSS Syntax for utility for CHU9D (Stevens, 2008). The measure was originally developed for use with ages 7-11 but it has since been validated for use with adolescents (Stevens and Ratcliffe, 2012). The CHU9D has also been demonstrated to have face, content, and construct validity (Stevens, 2011).

Child and Adolescent Service Use Schedule (CA-SUS; Byford et al., 2007)

Service use was recorded using a brief version of the "Child and Adolescent Service Use Schedule" (CA-SUS; Byford et al., 2007) in a format that was suitable for self-completion by adolescents in schools. The measure asked pupils to recall use of key services and resources over the last three months. Data were collected on the number of contacts with hospital services (e.g., inpatient stays, outpatient contacts, accident and emergency attendances), community health and social care services (e.g., GP, social worker, pharmacist, school nurse etc.), accommodation services (e.g., foster care, residential care, respite care), and teaching support services as well as prescribed psychotropic medication. The CA-SUS was based on previous versions that have been successfully applied in adolescent depression populations (Byford et al., 2007), in particular a brief version focused on key services (high cost and high volume of use) which was designed for self-completion by parents of primary school children in a school-based cluster RCT (Ford et al., 2018). Economic data were collected at pre- and

Kuyken W, *et al. Evid Based Ment Health* 2022; 25:99–109. doi: 10.1136/ebmental-2021-300396

post-intervention and at one-year follow-up. Reported service use at each timepoint was scaled to cover the entire duration of the follow-up period.

Sessions Attended

The number of SBMT sessions that pupils attended (which ranged between 0 and 10) was completed by the SBMT teacher teaching the lesson, who was in charge of taking pupil attendance.

Pupil acceptability

We assessed pupil's ratings of the SBMT's acceptability at post-intervention using a 5-item rating-scale measure ("How much does what's being taught in these lessons make sense to you in helping you to deal with issues young people face?", "Do you think that these lessons will help you have a healthier lifestyle?", "Would you recommend these lessons to a friend?", "How important do you think it is that these lessons are available to young people?", "How successful do you believe these lessons are likely to be in decreasing problems or issues that young people have?"), answered on a Likert-type scale (0 ="not at all", 10 ="a great deal"). Total scores were calculated by summing all the items divided by the number of items (mean total scores, ranging between 0 and 10). The internal consistency (Cronbach's alpha value) of this measure at post-intervention was $\alpha = 0.95$.

Pupil engagement

We assessed the extent (i.e., frequency) of pupil home-based mindfulness practice during the SBMT intervention, and also after the SBMT intervention, using a 6-item rating-scale measure that included the following items: "During the course you were taught a range of mindfulness practices. How often did you practice being mindful?", "During the course you were invited to pause and focus on your breathing by doing a 7-11 or FOFBOC or a .b (i.e. stop, breathe and be). How often did you do this?", "During the course you were taught to use 'beditation' as a way of helping you get to sleep. How often did you do this?", "During the course you were asked to be mindful in your everyday lives, for example walk a short distance mindfully, or eat a mouthful of food mindfully. How often did you do this?", "During the course you were asked to notice stress in your body, e.g. 'stress signature' in difficult times, noticing where in the body you were feeling stress. How often did you do this?", "During the course you were taught to think about your thoughts as passing objects such as buses, clouds or rivers that pass through your mind. How often did you do this?". Items were answered at post-intervention (to respond to the frequency of mindfulness practice during the SBMT intervention), and at 1-year follow up (to respond to the frequency of mindfulness practice after the SBMT intervention), on a Likert-type scale, ranging from 0 = "never", to 5 = "almost every day". Scores were calculated by summing all the items divided by the number of items (mean total scores, that ranged from 0 to 5). Therefore, higher scores represent a higher frequency of pupil home-based mindfulness practice. The internal consistency (Cronbach's alpha value) of this measure of pupil engagement with the mindfulness practice at post-intervention was $\alpha = 0{\cdot}89$, being of $\alpha = 0{\cdot}89$ at the 1-year follow up measurement.

Teacher Measures

Adherence (Fidelity)

The fidelity to the original ".b" SBMT programme was measured as the percentage of the standardised curriculum that was covered overall in two randomly selected lessons per intervention class. All SBMT lessons were filmed and a subset of 2 out of the 10 possible lessons from each class were evaluated. The two lessons evaluated for each class were randomly chosen by a computer random number generator from a subset of combinations which were chosen as they provided the best opportunities of observing a full practice (these possible combinations were: 3&6, 3&7, 4&6, where available; in cases where these classes were not available, e.g. not recorded, other appropriate classes were reviewed). Teachers did not know in advance that these combinations would be chosen. For each randomly selected lesson observed, independent evaluators indicated whether key curriculum elements (essential and non-essential, as they are defined by the ".b" SBMT teaching materials), were delivered or not. These ratings were summarised as the percentage of curriculum elements covered per each lesson, and they were finally averaged across the two ".b" randomly selected lessons to provide a percentage of elements covered per intervention class.

MBI-TAC-Teach - Competency (Quality)

To assess the quality of the SBMT intervention we considered the competency of the teaching that pupils received. All SBMT lessons were filmed and a randomly selected subset of 2 out of the 10 possible lessons from each class were rated using the "Mindfulness-Based Interventions – Teaching Assessment Criteria" (MBI-TAC; Crane et al., 2020). External evaluators did not see the pupils but only the research team, as the videos were anonymised. Lessons were rated by one of four different assessors using an adapted version of the MBI-TAC for the teaching context (MBI-TAC-Teach). External evaluators were experienced MBI teachers, who had a recognised mindfulness training pathway, were qualified to teach the SBMT (".b") having more than two years of experience, and were qualified classroom teachers. They were trained in the use of the MBI-TAC-Teach assessment taking part in two days of training where the MBI-TAC-Teach was introduced by two experienced supervisors. They all had experience of being rated by the MBI-TAC in their own teaching pathway so all of them were familiar with that tool. The training focused on the specific aspects of the MBI-TAC-Teach tool, and the training was based on collective discussions and evaluations of some case studies to ensure standardisation. Evaluators were also allowed time to rate some examples independently to ensure consistency and that these ratings were within an acceptable range for all the evaluators at the end of the training. All evaluators took part in regular supervision sessions with the aim of ensuring/maximising assessment standardisation. A randomly chosen 'back-up' lesson was also used by the evaluators if they felt that observing the first two lessons did not provide sufficient evidence for the overall ratings. Certain lessons were not used, for example, lesson 5 ('Moving Mindfully') as pupils move around in this lesson meaning that it was not easy to completely capture the pupil and teacher interactions on film. If videos were not available for the chosen combination of lessons, then a decision was made to use different lessons based upon the videos available and the lessons that would provide the best opportunity to observe all domains. The MBI-TAC was developed in the context of Mindfulness-Based Stress Reduction and Mindfulness-Based Cognitive Therapy and was adapted to MBI-TAC (Teach) to be used to rate classroom teachers teaching mindfulness to young people in school contexts. Competence is rated across 6 domains on a 6- point scale (1 = "incompetent", 2 = "beginner", 3 = "advanced beginner", 4 = "competent", 5 = "proficient", and 6 = "advanced"). The domains assess: coverage, pacing and organisation of session curriculum; relational skills; embodiment of

mindfulness; guiding mindfulness practices; conveying course themes through interactive enquiry and didactic teaching; and holding the group learning environment. Evaluators provided competency ratings on the 6 domains, and an overall competency rating per lesson (based on their own overall assessment rather than a sum score of the 6 domains), for the two randomly selected lessons per intervention class. Based on the two lessons, an overall rating per domain for that class was completed, and then used by evaluators to provide one overall final competency rating per class as a measure of the quality of the intervention delivery.

Implementation of mindfulness within the school curriculum

It was measured to what extent ".b" was: i) additive to the PSHE curriculum, so that it was unaffected (either ".b" did not take place in PSHE lessons, or if they did, PSHE curriculum content was either not removed, or taught elsewhere if removed); ii) substitutive, and therefore PSHE content was removed to make room for .b and it was not taught elsewhere; iii) partially additive/partially substitutive, and thus PSHE curriculum content was condensed to make room for ".b" content.

School Measures

Broad context, community, and operational features

We gathered school characteristics such as the region (England, Scotland, Wales, Northern Ireland), urbanity (urban, rural), school size (<1,000 pupils, ≥1,000 pupils), type of school (mixed, girls only), Ofsted school quality rating (does not require improvement: outstanding, good; requires improvement: requires improvement, inadequate), school deprivation (% of pupils eligible for free school meals). We also described (a) the provision quality of 'Personal, Social, Health and Economic Education' (PSHE) (see next paragraph).

School quality is measured differently in public and private schools and across the nations. We developed a measure to allow all the different school inspection rating systems to map onto each other to allow us to use the terms "outstanding" to "requires improvement."

Social Emotional Learning (SEL) in England is taught as part of 'Personal, Social, Health and Economic Education' (PSHE) lessons. Due to the fact that delivering PSHE lessons in schools is not mandatory in England, there is a wide variation across schools in the delivery of PSHE lessons (in terms of content covered and teaching time allocated). A literature review highlighted that there was no existing measures of PSHE which would allow the current study to assess which schools had a minimum level of good practice in PSHE to be considered for participation in the study. Thus, a new PSHE assessment tool was devised for the current study. For inclusion in the trial, schools had to meet 5 criteria for their current PSHE provision: regular, discrete, named teaching time for PSHE (or equivalent); a designated PSHE lead; a named member of the Senior Leadership Team (SLT) responsible for PSHE; documentation denoting clear strategic planning of SEL within the school; and evaluation of pupil progress in PSHE. Once schools became a participating trial school, PSHE was assessed by discussing PSHE provision with the teacher responsible for PSHE at each school (or a member of the Senior Leadership Team). Sixteen quality indicators (listed below) were used to assess PSHE provision. They were created specifically for this trial and identified through a review of existing measures and via expert consultation (Department of

Education, 2010). Schools were assigned a score (out of 16) reflecting the number of quality indicators present (subscale scores indicate quality in the domains of Leadership and Strategic Approaches to PSHE, Curriculum Content and Delivery and Assessment, Evaluation, and Consultation). Total scores were used in the present study. The items used organised by sub-scales were the following:

Sub-scales	Indicators (score)
Leadership and Strategic Approaches to PSHE from Consensus Indicators	A designated PSHE lead (0 = no, 1 = yes)<br><br>A named member of SLT has responsibility for supporting PSHE (0 = no, 1 = yes)<br><br>A written PSHE policy (0 = no, 1 = yes)<br><br>School's own rating of the quality of its PSHE provision (0-4 = 0, 5-10 = 1)<br><br>PSHE provision is part of the school improvement plan (0 = no, 1 = yes)<br><br>How well-informed does PSHE lead feel about local PSHE education CPD opportunities (0-4 = 0, 5-10 = 1)
Curriculum Content and Delivery from Consensus Indicators	Regular discrete, named teaching time for PSHE, including drop down days or tutorial time (0 = no, 1 = yes)<br><br>PSHE lead teaches PSHE lessons (0 = no, 1 = yes)<br><br>Topic Coverage KS3 and KS4 - School provides coverage of all elements of PSHE curriculum (0 = no, 1 = yes)<br><br>PSHE lead involved in planning: evidence of attempts to plan and coordinate PSHE across KS3 and KS4 (0 = no, 1 = yes)<br><br>Teaching Methods Used: School uses at least 6/10 methods for delivering PSHE (0 = no, 1 = yes)
Methods of Assessment, Evaluation and Consultation from Consensus Indicators	Any evaluation of pupil progress in PSHE (0 = no, 1 = yes)<br><br>Informal feedback (0 = no, 1 = yes)<br><br>Pupil / peer assessment of feedback (0 = no, 1 = yes)<br><br>Written feedback on pupil progress reports (0 = no, 1 = yes)<br><br>School uses feedback to plan PSHE (0 = no, 1 = yes)

Kuyken W, *et al. Evid Based Ment Health* 2022; 25:99–109. doi: 10.1136/ebmental-2021-300396

Supplement C

Costs

For each item of service use reported in the CA-SUS, a nationally applicable unit cost was applied to calculate the costs for each participant. Unit costs for hospital services were sourced from NHS reference costs (Department of Health, 2019). Costs from the annually published unit costs of health and social care compendium were applied to community-based health, social care and Local Authority accommodation services (Curtis & Burn, 2019). Costs applied to some community services were also sourced from webpages of charitable organisations (NSPCC, 2021). The costs of medications were based on prices listed in the British National Formulary for Children (Royal Pharmaceutical Society of Great Britain, 2019) and Prescription Cost Analysis data (NHS Business Services Authority Statistics, 2019). Costs for teaching support were estimated from information published by the National Education Union (National Education Union, 2019). All unit costs applied were for the financial year 2018-19 (summarised in Table S1) and are reported in UK pounds sterling. Costs incurred after 12-months from the start of the trial (for those whose 1-year follow-up was late) were discounted by 3.5%, as recommended by NICE (National Institute for Health and Clinical Excellence, 2013).

Resource inputs into SBMT training and delivery were costed using a micro-costing approach and included costs of training, materials, supply cover and subsistence. Data on intervention contacts and the costs of training and materials were collected directly from trial records. Total costs were calculated separately for phase 1 (self-mindfulness training) and phase 2 (syllabus training) for all teachers taking part in each phase of the trial.

Supplementary Table S1: Unit costs and sources

Service (unit)	Unit cost	Source	Notes
**Hospital services**			
Inpatient - mental health (night)	802·00	1	Weighted average all paediatric admission activity relating to mental health
Inpatient - injury (night)	552·00	1	Weighted average of short-stay paediatric admission activity based on categories labelled as injury
Inpatient - anything else (night)	517·00	1	Weighted average of all other short-stay paediatric admission activity
Outpatient - mental health (attendance)	242·00	1	Weighted average of all paediatric non-admission activity relating to mental health
Outpatient - injury (attendance)	126·00	1	Weighted average of paediatric non-admission activity relating to trauma, orthopaedics and burns
Outpatient - anything else (attendance)	207·00	1	Weighted average of all other paediatric non-admission activity
Accident and emergency (attendance)	166·00	1	Weighted average of all activity in paediatric A&E
Ambulance (attendance)	257·00	1	See and treat and convey ambulatory activity
**Community services**			
GP (contact)	39·23	2	Based on per patient contact lasting 9.22 minutes
Nurse or midwife (contact)	21·00	2	Based on per patient contact with nurse in GP practice
CAMHS (contact)	234·00	2	Average cost per patient contact in community setting
Social worker (contact)	25·00	2	Based on per patient contact lasting 30 minutes
Pharmacist (contact)	7·50	2	Based on per patient contact with scientific and professional staff (band 6) lasting 10 minutes
School nurse (contact)	59·00	2	Based on per pupil contact with one-to-one school-based children's core health services
School counsellor/education psychologist (contact)	47·00	2	Proxy used - counselling for children with mental or emotional difficulties; based on average cost for client-related activity lasting for 30 minutes
Speech and language therapist (contact)	99·00	2	Based on per patient contact with one-to-one services lasting 60 minutes
Counselling (contact)	78·33	2	Based on average cost for client-related activity lasting 50 minutes
Other therapy e.g., art/music/drama therapy (contact)	123·50	2	Based on average cost for one-to-session with physio and occupational therapist
Helplines (contact)	4·50	3, 4	Based on average cost per call for Childline and Samaritans

**Medication**			
Depression, anxiety, eating disorders (day)	0·09	5, 6	Based on average costs of fluoxetine and citalopram administered as per BNFC guidelines
ADHD (day)	1·11	5, 6	Based on costs of methylphenidate administered as per BNFC guidelines
Sleep disorders (day)	1·27	5, 6	Based on average costs of melatonin and Valium administered as per BNFC guidelines
Tics/Tourette's (day)	0·27	5, 6	Based on market share and costs of risperidone and haloperidol administered as per BNFC guidelines
Psychosis (day)	0·27	5, 6	Based on market share and costs of sulpiride, aripiprazole, risperidone, haloperidol administered as per BNFC guidelines
**Teaching/learning support**			
Used sometimes (day)	3·87	2, 7	Based on one-to-one contact with level TA4 for 1 hour per week
Used often (day)	19·33	2, 7	Based on one-to-one contact with level TA4 for 5 hours per week
**Accommodation services**			
Foster care (day)	88·71	2	Based on care provided by Local Authority, excluding social care support directly related to fostered children but including additional education services
Residential care/children's home (day)	677·00	2	Based on establishment costs per resident day, excluding other costs such as school support
Respite care (day)	88·71	2	Proxy used - foster care provided by Local Authority

Sources: 1National Schedule of Unit Costs, 2 Unit Costs of Health and Social Care 2019, 3 Childline webpages, 4 Samaritans webpages, 5 BNF for Children, 6 Prescription Cost Analysis - England 2019, 7 National Education Union

Supplement D

Further details of economic methods

The use of all services is reported by trial arms as the mean, standard deviation, and range as well as the percentage of the sample for each arm that had at least one contact. Differences in resource use were not tested for statistical significance to avoid excessive significance testing and to keep the focus of the economic analysis on cost and cost-effectiveness.

For each participant, all costs were summed to calculate total costs over the 1-year follow-up. Costs and outcomes, including costs per sector, were summarised using the mean and standard error for each trial arm and the differences between the two were compared using standard parametric t-tests. Despite the skewed nature of cost data, this method allows inferences to be made about the arithmetic mean, which are more meaningful from a decision-making perspective (Thompson & Barber, 2000).

Cost-effectiveness was assessed using the net benefit approach (Stinnett & Mullahy, 1998), with joint effects of costs and outcomes estimated using mixed effects linear regression. A cost-effectiveness acceptability curve (CEAC) (Fenwick & Byford, 2005) was then constructed to examine the probability of the SBMT intervention being cost-effective compared to control for a range of possible values of willingness-to-pay per unit improvement in outcome. Cost-effectiveness was assessed first in terms of QALYs measured using the CHU9D (scenario #1). Secondary analyses explored cost-effectiveness in terms of the three co-primary clinical outcomes to assess the sensitivity of analyses to the alternative outcomes of interest (scenarios #2-4).

Pre-specified sensitivity analyses were undertaken to assess the impact of missing data by undertaking a complete-case analysis (scenario #5), the impact of taking a health and social care perspective preferred by NICE (excluding teaching support; scenario #6) and including only mental health services to better explore the impact of the intervention on mental health (scenario #7).

Two post-hoc sensitivity analyses were added. First, due to difficulties in timetabling around school holidays, four schools (three in intervention, one in control) completed the 1-year follow-up assessment after 14-months post-randomisation (Figure S1). We therefore assessed the impact of variable follow-up duration by excluding cases with follow-up assessment after 14-months post-randomisation (scenario #8). Secondly, reports of use of prescribed psychotropic medication were deemed unreliable as a result of reports of use of anti-psychotic medication that were substantially higher than general population prescribing patterns. We therefore assessed the impact of the quality of this data by excluding service use data on the use of prescribed psychotropic medication (scenario #9).

Supplement E

Further details on the CONSORT diagram

The baseline characteristics of the schools and pupils have previously been reported (Ford et al., 2021). Randomisation of schools (clusters) was followed by 'randomisation of study classes' within schools which happened over the space of approximately six months at maximum, up until the pre-intervention data collection. Discussions with schools centred on approximate classes, often using class identifiers which at the time did not specifically refer to particular pupils. Rather, class identifiers were a proxy for pupils.

Pupils only became 'study pupils' at the point at which we received and confirmed who was in the selected study classes (i.e., confirmed the registers). For some schools, this was on our pre-intervention visit. Therefore, individual pupils did not have the opportunity to 'drop out' of the study between randomisation of schools, randomisation of study classes and pre-intervention data collection, as the pupils were 'selected' up until that point.

Kuyken W, *et al. Evid Based Ment Health* 2022; 25:99–109. doi: 10.1136/ebmental-2021-300396

Supplement F

Representativeness of study schools and pupils

Eighty seven percent of schools were mixed (the remainder being girls only schools); 13% required improvement based on their school quality rating (for schools outside England, they were reviewed and scored as per the England ratings to align to the same scale); and just over a third of schools had a higher percentage of children eligible for free school meals than the national median (29·4%) (Department of Education, 2020).

	**Number of schools**	**Target (number of schools)/85 (Based on national average)**	**Notes**
**Country**			
England	75	68	Slightly more schools in England, under for Wales and Scotland, but exactly as per target in Northern Ireland
Wales	3	5	
Scotland	3	8	
Northern Ireland	4	4	
**Gender**			
Single sex	12	9	A few more single sex schools, and all female schools. No all boys schools.
Mixed	73	77	
**Deprivation (FSM in last 6 years)**			
Above average	29	21	Slightly more deprived schools in the study than the national average.
Below average	56	64	
**Quality Measure**			
Outstanding/Excellent/Very good	13	20	Many new schools or new academy converter schools were not yet quality rated by the national body (OFSTED). Most of our schools in the 'good' category
Good/satisfactory	40	48	
Requires improvement/adequate	13	14	
Not yet rated/NA/UNK	19	4	
**Type of school**			
Selective	8	4	Slightly more selective, and slightly less independent, but close to target numbers
Independent	3	7	
**Size of school:**			
Small (<1000)	39	45	Good split of small vs large schools
Large (>1000)	46	40	

Kuyken W, *et al. Evid Based Ment Health* 2022; 25:99–109. doi: 10.1136/ebmental-2021-300396

Descriptive data at baseline and pre-intervention suggest we broadly recruited a sample of pupils who are representative with respect to our primary outcomes regarding risk for depression (mean (SD) was 13·9 (9·7) in a previous study of Canadian students aged 12 or 13) (Briere et al., 2013), and well-being (mean (SD) was 48·8 (6·8) in a previous study of UK pupils aged 13 to 16) (Clarke, et al., 2011) but had slightly poorer social-emotional behavioural functioning (normative mean (SD) was reported to be 10·3 (5·2) on SDQ information website accessed on 26th April 2021: https://www.sdqinfo.org/norms/UKNorm3.pdf) than the wider population.

All school level measures (e.g. Admissions type, Urbanicity and school size) were obtained using publicly available data published by the constituent nation the participating schools resided within. Data was usually obtained online from the education and statistics departments (e.g., Department of Education, England). Where this wasn't available, the data was obtained through email correspondence with the department. In all cases, published publicly available data were collected according to its proximity to the year in which participating pupils provided Baseline (T0) questionnaire data. Specifically, characteristics of the school community and operational features of the school were all obtained using data collected and released by the government as part of the annual school census. All public schools in the UK are required to provide data to their local authority yearly, which is then processed and released by the relevant nations governing education or statistical department.'

School rating: Each country within the UK has its own schools inspectorate, each with a different quality rating system. To allow for analysis of school quality ratings across schools we mapped them all onto a single quality rating system. The majority of the schools in the project are inspected by Ofsted (England's state-funded schools inspectorate) with only 12 schools evaluated by a different inspectorate. It was therefore decided the 12 schools would have their ratings mapped onto the Ofsted rating system. Two researchers independently analysed the 12 schools' inspectorate reports and assigned them a quality rating from 1 – 4 to be in line with Ofsted quality ratings (1 being the highest quality rating and 4 the lowest). None of the schools were given a rating of 4 (inadequate), this is in-line with the exclusion criteria for project participation which stated that to take part a school could not have an inspection rating of inadequate or equivalent. The report published closest to, and before, the date the school entered the project was used where possible. The Ofsted School Inspection Handbook 2018 informed the researchers' mappings. The inter-rater reliability for mappings was 91%.

These are the websites from which data was sourced:

National average free school meals of 13.2%:
https://www.gov.uk/government/uploads/system/uploads/attachment_data/file/552342/SFR20_2016_Main

Deprivation:
Free school meals - eligible in last 6 years = 29.4%
https://www.compare-school-performance.service.gov.uk/

Size of school
Size of school - cut off = 1000. Number taken from school census Jan 2016. Small <1000, Large >= 1000.
1 school were data not available from school census, total taken from Gov.uk

http://www.education.gov.uk/edubase/home.xhtml
https://www.compare-school-performance.service.gov.uk/

OFSTED rating:
https://reports.ofsted.gov.uk/

Supplement G

Frequencies of pupil's ratings of the SBMT's acceptability

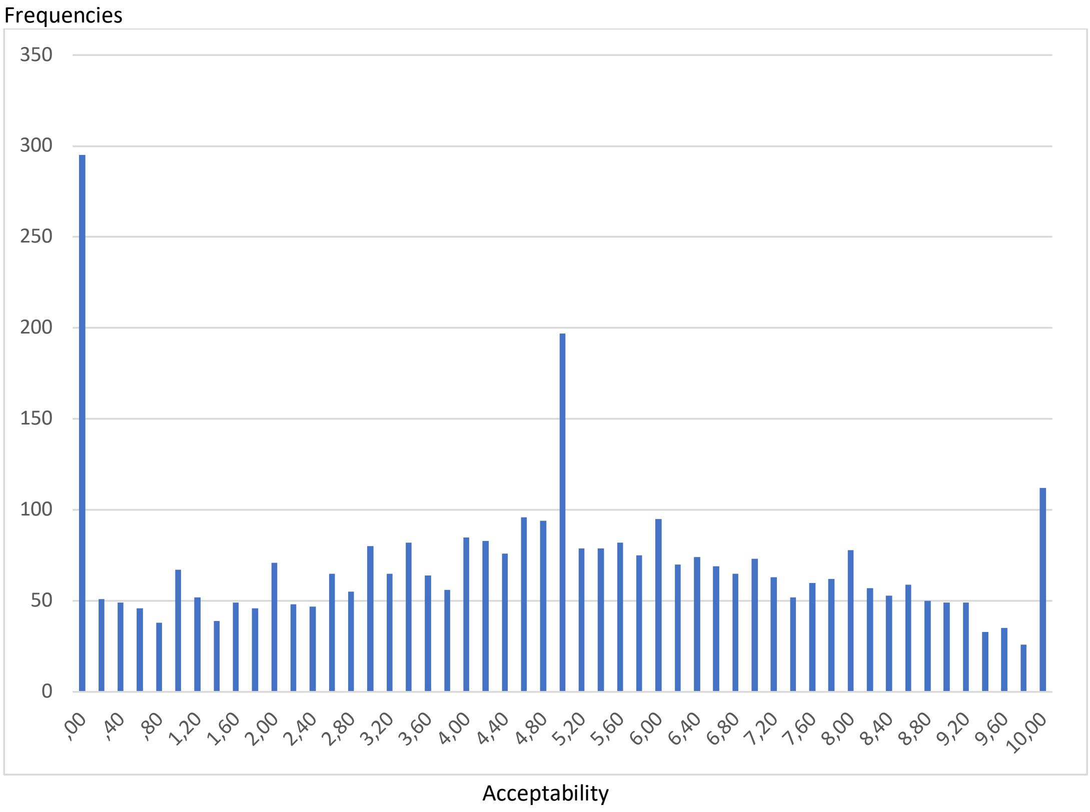

N valid = 3,595. Missing values = 637. Mean = 4·7. SD = 2·9.

Kuyken W, *et al. Evid Based Ment Health* 2022; 25:99–109. doi: 10.1136/ebmental-2021-300396

Supplementary Table S2: Selected study/trial pupil baseline characteristics by 1 year follow-up status

	Pupils lost to follow-up*			Remaining pupils**		
**Variables**	**Intervention arm (n = 554)**	**Control arm (n = 572)**	**Total (n = 1,126)**	**Intervention arm (n = 3,678)**	**Control arm (n = 3,572)**	**Total (n = 7,250)**
Gender						
Female, n (%)	279 (52·1)	283 (50·2)	562 (51·1)	2,071 (57·2)	1,876 (53·6)	3,947 (55·4)
Male, n (%)	243 (45·3)	266 (47·2)	509 (46·3)	1,481 (40·9)	1,557 (44·5)	3,038 (42·7)
Other, n (%)	5 (0·9)	2 (0·4)	7 (0·6)	9 (0·3)	10 (0·3)	19 (0·3)
Prefer not to say, n (%)	9 (1·7)	13 (2·3)	22 (2·0)	60 (1·7)	56 (1·6)	116 (1·6)
Ethnicity – White†, n (%)	404 (75·8)	420 (74·7)	824 (75·3)	2,833 (78·4)	2,545 (73·0)	5,378 (75·8)
Age, mean (SD)	12.2 (0.6)	12.2 (0.6)	12.2 (0.6)	12.2 (0.6)	12.2 (0.6)	12.2 (0.6)
Starting Year group						
Year 7, n (%)	292 (52·7)	283 (49·5)	575 (51·1)	1,790 (48·7)	1,859 (52·0)	3,649 (50·3)
Year 8, n (%)	245 (44·2)	280 (49·0)	525 (46·6)	1,633 (44·4)	1,547 (43·3)	3,180 (43·9)
Year 9, n (%)	0 (0·0)	7 (1·2)	7 (0·6)	79 (2·2)	57 (1·6)	136 (1·9)
Year S1, n (%)	17 (3·1)	2 (0·4)	19 (1·7)	176 (4·8)	109 (3·1)	285 (3·9)
Depression (CES-D)††, mean (SD)	16.3 (10·9)	14.7 (10·6)	15.5 (10·7)	13.2 (9·7)	13.1 (9·7)	13.1 (9·7)
Social/emotional/behavioural functioning (SDQ) Total Difficulties – self report†††, mean (SD)	13.8 (6·7)	13.2 (6·5)	13.5 (6·6)	11.5 (6·5)	11.5 (6·3)	11.5 (6·4)
Well-being (WEMWBS)††††, mean (SD)	47.8 (10·1)	48.7 (10·4)	48.2 (10·2)	50.0 (9·6)	49.8 (9·6)	49.9 (9·6)

* Defined as those pupils with missing data on all 3 co-primary outcomes at 1 year follow-up.
** Defined as those pupils with at least one of the co-primary outcomes at 1 year follow-up.
† Sample size in lost to follow-up group: 1,095: intervention arm: 533; control arm: 562. Sample size in remaining pupils group: 7,098: intervention arm: 3,612; control arm: 3,486
†† Sample size in remaining pupils group: 7,244: intervention arm: 3,676; control arm: 3,568
††† Sample size in lost to follow-up group: 1,104: intervention arm: 539; control arm: 565. Sample size in remaining pupils group: 7,148: intervention arm: 3,632; control arm: 3,516.
†††† Sample size in lost to follow-up group: 1,120: intervention arm: 549; control arm: 571. Sample size in remaining pupils group: 7,213: intervention arm: 3,665; control arm: 3,548.
CES-D: Center for Epidemiologic Studies for Depression Scale. SDQ: Strengths and Difficulties Questionnaire. WEMWBS: Warwick-Edinburgh Mental Well-Being Scale.

Supplementary Table S3: Main comparisons of student outcomes at post-intervention follow-up: modified intent-to-treat (complete case) analysis

Outcome	Intervention arm (I)		Control arm (C)		Unadjusted	Adjusted mean difference (I-C) / odds ratio (I/C)[a]			ICC[b]
	n	mean (SD) / n (%)	n	mean (SD) / n (%)	mean diff (I-C) / odds ratio (I/C)[a]	estimate	95% CI	p-value	
Depression (CES-D)	3,768	16·9 (11·8)	3,793	16·4 (11·6)	0·3	0·1	-0·5 to 0·7	0·76	0·019
Social/emotional/behavioural functioning (SDQ) – self report									
Total Difficulties	3,752	13·4 (6·9)	3,790	13·1 (6·8)	0·2	0·1	-0·2 to 0·5	0·40	0·020
Well-being (WEMWBS)	3,775	47·8 (9·6)	3,797	48·1 (9·3)	-0·2	-0·1	-0·6 to 0·4	0·71	0·017
Social/emotional/behavioural functioning (SDQ) – self report									
Emotional Symptoms	3,752	4·0 (2·8)	3,790	3·8 (2·7)	0·1	0·1	-0·1 to 0·2	0·34	0·022
Conduct Problems	3,752	2·6 (2·0)	3,790	2·6 (2·0)	-0·02	-0·02	-0·1 to 0·1	0·65	0·014
Hyperactivity/Inattention	3,752	4·7 (2·6)	3,790	4·5 (2·6)	0·1	0·1	-0·001 to 0·3	0·05	0·015
Peer Relationship Problems	3,752	2·2 (1·9)	3,790	2·2 (1·9)	-0·01	-0·02	-0·1 to 0·1	0·69	0·013
Prosocial behaviour	3,752	7·5 (2·0)	3,790	7·4 (1·9)	0·02	-0·04	-0·1 to 0·1	0·44	0·020
Executive processing (BRIEF2) – self-report	3,115	85·7 (22·2)	3,426	84·9 (21·9)	0·5	0·02	-1·7 to 1·7	0·99	0·015
Executive processing (BRIEF2) – teacher-report	3,083	80·2 (24·6)	2,451	80·0 (24·5)	-1·1	-1·0	-5·1 to 3·0	0·63	0·098
Drug use – self-report	3,410	458 (13·4)	3,621	524 (14·5)	0·9	0·9	0·8 to 1·1	0·39	0·015
Alcohol use – self-report	3,428	1,556 (45·4)	3,630	1,752 (48·3)	0·9	0·9	0·7 to 1·1	0·37	0·081
Anxiety (RCADS) – self-report									
Total score	3,487	31·3 (21·6)	3,688	29·1 (20·8)	1·8	1·2	-0·3 to 2·7	0·13	0·028
Social phobia	3,492	10·9 (6·9)	3,694	10·4 (6·6)	0·4	0·2	-0·2 to 0·7	0·32	0·040

Kuyken W, *et al. Evid Based Ment Health* 2022; 25:99–109. doi: 10.1136/ebmental-2021-300396

Panic disorder	3,487	6·5 (6·1)	3,689	5·8 (5·9)	0·6	0·4	-0·02 to 0·8	0·06	0·020
Separation anxiety	3,491	3·4 (3·6)	3,693	3·1 (3·5)	0·2	0·2	-0·1 to 0·4	0·22	0·019
Generalized anxiety	3,499	6·2 (4·5)	3,697	5·8 (4·3)	0·3	0·2	-0·1 to 0·5	0·22	0·027
Obsessive-compulsive	3,496	4·4 (3·8)	3,695	4·1 (3·7)	0·3	0·3	-0·02 to 0·5	0·07	0·018
Social/emotional/behavioural functioning (SDQ) – teacher-report									
Total Difficulties	3,071	5·9 (6·0)	2,451	5·6 (5·8)	0·2	0·1	-0·7 to 0·9	0·82	0·054
Emotional Symptoms	3,071	1·2 (1·9)	2,451	1·0 (1·9)	0·1	0·1	-0·2 to 0·4	0·47	0·043
Conduct Problems	3,071	0·9 (1·7)	2,451	0·8 (1·5)	0·1	0·1	-0·1 to 0·2	0·43	0·012
Hyperactivity/Inattention	3,071	2·5 (2·7)	2,451	2·5 (2·7)	-0·04	-0·1	-0·4 to 0·3	0·74	0·047
Peer Relationship Problems	3,071	1·3 (1·7)	2,451	1·3 (1·7)	0·03	0·01	-0·2 to 0·2	0·90	0·030
Prosocial behaviour	3,071	7·2 (2·6)	2,451	7·2 (2·6)	0·1	0·03	-0·3 to 0·4	0·88	0·039
Self-harm – self-report	3,389	365 (10·8)	3,431	361 (10·5)	1·0	1·0	0·8 to 1·2	0·82	0·012
Suicide ideation – self-report	3,251	745 (22·9)	3,246	718 (22·1)	1·0	1·0	0·8 to 1·2	0·93	0·016
Mindfulness skills (CAMM) – self-report	3,703	26·0 (8·3)	3,769	26·7 (8·3)	-0·6	-0·5	-1·1 to 0·1	0·08	0·024
School ecology/climate (SCCS)									
School Leadership and Student Involvement	3,606	3·2 (0·8)	3,749	3·2 (0·9)	0·01	0·02	-0·1 to 0·1	0·67	0·072
Respectful Climate	3,600	3·2 (0·8)	3,746	3·3 (0·8)	-0·04	-0·02	-0·1 to 0·1	0·62	0·050
Peer Climate	3,598	3·0 (0·8)	3,745	3·0 (0·8)	-0·01	-0·03	-0·1 to 0·1	0·53	0·062
Caring Adults	3,593	3·1 (0·9)	3,744	3·2 (0·9)	-0·04	-0·02	-0·1 to 0·1	0·62	0·037

[a] Mean difference reported for quantitative outcomes and odds ratios reported for binary outcomes.
[b] Intra-cluster (intra-school) correlation coefficients (ICCs) from crude (unadjusted) analyses.
CES-D: Center for Epidemiologic Studies for Depression Scale. SDQ: Strengths and Difficulties Questionnaire. WEMWBS: Warwick-Edinburgh Mental Well-Being Scale. CAMM: Child and Adolescent Mindfulness Measure. BRIEF2: Behaviour Rating Inventory of Executive Function version 2. RCADS: Revised Child Anxiety and Depression Scale. SCCS: School Climate and Connectedness Survey.

Kuyken W, *et al. Evid Based Ment Health* 2022; 25:99–109. doi: 10.1136/ebmental-2021-300396

Supplementary Table S4: Main comparisons of student outcomes at 1 year follow-up: modified intent-to-treat (complete case) analysis

Outcome	Intervention arm (I)		Control arm (C)		Unadjusted	Adjusted mean difference (I-C) / odds ratio (I/C)[a]			ICC[b]
	n	mean (SD) / n (%)	n	mean (SD) / n (%)	mean diff (I-C) / odds ratio (I/C)[a]	estimate	95% CI	p-value	
Depression (CES-D)	3,672	17·1 (11·9)	3,566	16·6 (11·9)	0·3	0·1	-0·6 to 0·7	0·81	0·019
Social/emotional/behavioural functioning (SDQ) – self report									
Total Difficulties	3,664	13·2 (6·8)	3,561	12·9 (6·8)	0·2	0·2	-0·1 to 0·5	0·26	0·017
Well-being (WEMWBS)	3,678	47·6 (9·8)	3,566	47·6 (9·8)	0·1	0·2	-0·4 to 0·7	0·55	0·016
Social/emotional/behavioural functioning (SDQ) – self report									
Emotional Symptoms	3,664	4·0 (2·7)	3,562	3·8 (2·7)	0·1	0·1	-0·05 to 0·2	0·25	0·020
Conduct Problems	3,664	2·4 (2·0)	3,562	2·5 (2·0)	-0·03	-0·05	-0·1 to 0·1	0·36	0·011
Hyperactivity/Inattention	3,664	4·6 (2·6)	3,562	4·5 (2·5)	0·2	0·2	0·05 to 0·3	0·01	0·013
Peer Relationship Problems	3,664	2·2 (1·9)	3,561	2·2 (1·9)	-0·02	-0·01	-0·1 to 0·1	0·82	0·015
Prosocial behaviour	3,664	7·4 (2·0)	3,562	7·4 (2·0)	0·04	-0·02	-0·1 to 0·1	0·76	0·022
Executive processing (BRIEF2) – self-report	3,288	84·3 (22·7)	3,329	83·6 (22·4)	0·5	0·04	-1·6 to 1·6	0·96	0·021
Executive processing (BRIEF2) – teacher-report	2,489	77·8 (22·8)	1,990	78·7 (24·3)	-1·7	-1·0	-5·6 to 3·6	0·67	0·102
Drug use – self-report	3,401	587 (17·3)	3,429	635 (18·5)	0·9	0·9	0·8 to 1·1	0·38	0·017
Alcohol use – self-report	3,436	1,703 (49·6)	3,451	1,729 (50·1)	1·0	1·0	0·8 to 1·2	0·91	0·092
Anxiety (RCADS) – self-report									
Total score	3,504	30·0 (21·5)	3,483	28·8 (21·6)	0·9	0·3	-1·3 to 1·8	0·73	0·028
Social phobia	3,510	10·6 (6·9)	3,488	10·3 (6·8)	0·1	-0·1	-0·5 to 0·4	0·80	0·033
Panic disorder	3,504	6·2 (6·1)	3,485	5·8 (6·1)	0·4	0·2	-0·2 to 0·6	0·41	0·023

Kuyken W, *et al. Evid Based Ment Health* 2022; 25:99–109. doi: 10.1136/ebmental-2021-300396

Separation anxiety	3,508	3·2 (3·5)	3,488	3·1 (3·6)	0·1	0·06	-0·2 to 0·3	0·63	0·016
Generalized anxiety	3,512	5·9 (4·4)	3,490	5·7 (4·4)	0·1	-0·01	-0·3 to 0·3	0·93	0·026
Obsessive-compulsive	3,512	4·1 (3·7)	3,489	3·9 (3·8)	0·2	0·1	-0·2 to 0·4	0·51	0·017
Social/emotional/behavioural functioning (SDQ) – teacher-report									
Total Difficulties	2,496	5.3 (5·7)	1,981	5·1 (5·8)	-0·1	0·1	-0·9 to 1·1	0·80	0·075
Emotional Symptoms	2,496	1·1 (1·9)	1,981	1·0 (1·8)	0·1	0·2	-0·1 to 0·5	0·19	0·051
Conduct Problems	2,496	0·7 (1·5)	1,981	0·8 (1·5)	-0·1	-0·03	-0·2 to 0·2	0·76	0·031
Hyperactivity/Inattention	2,496	2·2 (2·6)	1,981	2·2 (2·6)	-0·1	-0·03	-0·4 to 0·4	0·87	0·055
Peer Relationship Problems	2,496	1·2 (1·7)	1,981	1·2 (1·7)	-0·04	-0·002	-0·3 to 0·3	0·99	0·043
Prosocial behaviour	2,496	7·6 (2·5)	1,981	7·5 (2·6)	0·3	0·2	-0·3 to 0·7	0·41	0·092
Self-harm – self-report	3,364	389 (11·6)	3,234	385 (11·9)	1·0	0·9	0·8 to 1·1	0·47	0·008
Suicide ideation – self-report	3,224	779 (24·2)	3,098	709 (22·9)	1·1	1·1	0·9 to 1·2	0·51	0·015
Mindfulness skills (CAMM) – self-report	3,625	26·4 (8·5)	3,546	26·7 (8·7)	-0·3	-0·02	-0·6 to 0·5	0·95	0·020
School ecology/climate (SCCS)									
School Leadership and Student Involvement	3,587	3·1 (0·9)	3,530	3·1 (0·9)	-0·02	-0·01	-0·1 to 0·1	0·89	0·067
Respectful Climate	3,582	3·2 (0·8)	3,527	3·2 (0·8)	-0·01	-0·01	-0·1 to 0·1	0·88	0·035
Peer Climate	3,581	3·0 (0·7)	3,523	3·0 (0·7)	-0·001	-0·01	-0·1 to 0·1	0·78	0·057
Caring Adults	3,576	3·1 (0·9)	3,518	3v2 (0·9)	-0·04	-0·03	-0·1 to 0·04	0·47	0·032

[a] Mean difference reported for quantitative outcomes and odds ratios reported for binary outcomes.
[b] Intra-cluster (intra-school) correlation coefficients (ICCs) from crude (unadjusted) analyses.
CES-D: Center for Epidemiologic Studies for Depression Scale. SDQ: Strengths and Difficulties Questionnaire. WEMWBS: Warwick-Edinburgh Mental Well-Being Scale. CAMM: Child and Adolescent Mindfulness Measure. BRIEF2: Behaviour Rating Inventory of Executive Function version 2. RCADS: Revised Child Anxiety and Depression Scale. SCCS: School Climate and Connectedness Survey.

Supplement H

Details of all economic analyses

Length of 1-year follow-up across trial arms

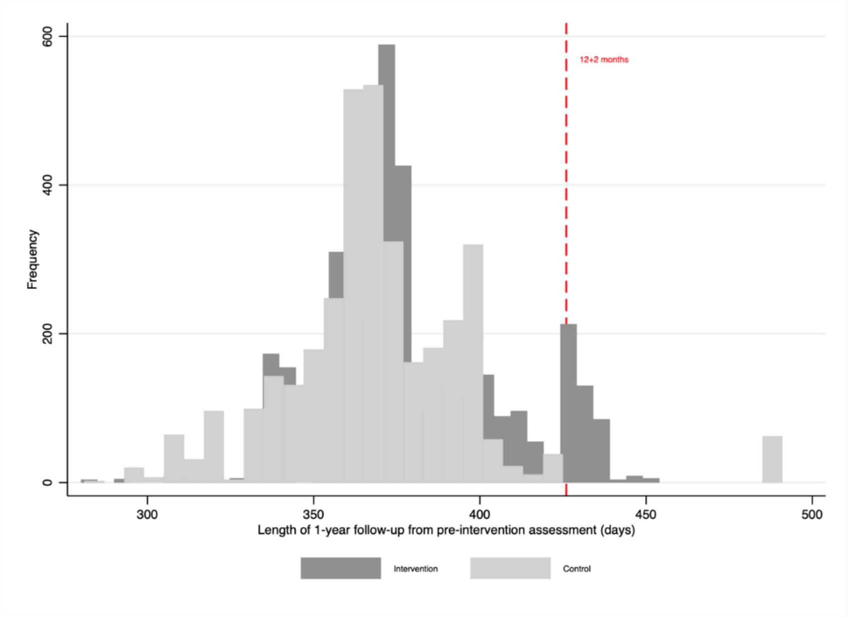

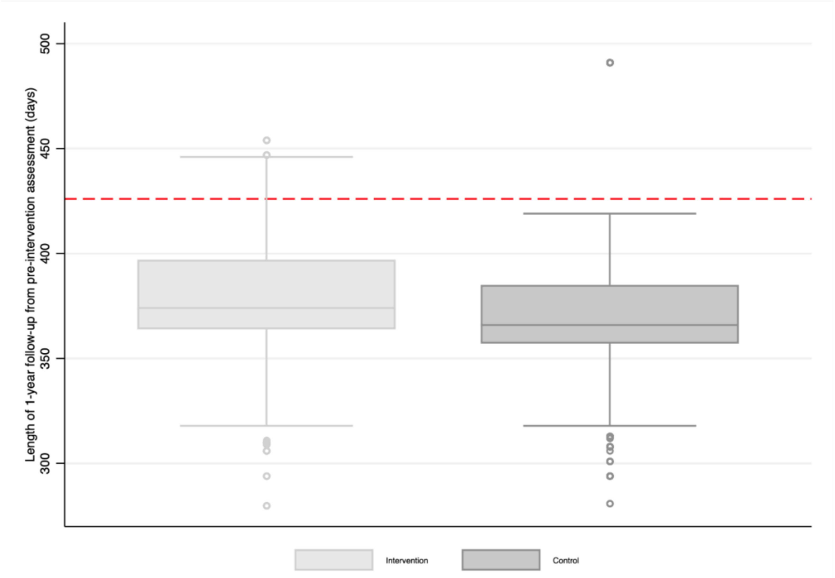

Availability of data

The availability of health economics data is summarised in Supplementary Table S5. Data for all assessment periods were available for 3313 (78%) participants in the intervention group and 3287 (79%) participants in the control group.

Supplementary Table S5: Availability of health economics data

	**Intervention n (%)**	**Control n (%)**
Pre-intervention service use	4080 (96%)	3995 (96%)
Pre-intervention CHU9D utility	4029 (95%)	3953 (95%)
Post-intervention service use	3790 (90%)	3804 (92%)
Post-intervention CHU9D utility	3736 (88%)	3779 (91%)
1-year follow-up service use	3690 (87%)	3579 (86%)
1-year follow-up CHU9D utility	3651 (86%)	3551 (86%)
All data	3313 (78%)	3287 (79%)
All data excl. outliers	3265 (77%)	3231 (78%)

Service use

Supplementary Table S6 summarises the use of hospital, community health and social care, accommodation, and teaching support services over the 1-year follow-up period. Overall, all services were accessed by similar proportions of participants and mean service use was also similar across trial arms.

For hospital services, mean service use for highest for outpatient appointments related to injury for both trial arms (mean number of appointments 1.38 in intervention and 1.34 in control). Mean service use was low for attendances related to mental health problems for both inpatient (mean number of nights 0.04 in intervention and 0.03 in control) and outpatient (mean number of appointments 0.33 in intervention and 0.29 control) services. GP, pharmacist, and school nurse were the most commonly used community services in both trial arms. Less than 1% of participants used Local Authority provided accommodation (foster or residential care) across trial arms. Use of teaching support was the same across trial arms (14% of participants used teaching support on some days and 2% used teaching support daily).

Mean use was higher in the intervention group for some services (e.g., outpatient – other, nurse/midwife) and higher in the control group for others (e.g., counselling, respite care). Overall, however, these differences were very small.

Kuyken W, *et al. Evid Based Ment Health* 2022; 25:99–109. doi: 10.1136/ebmental-2021-300396

Supplementary Table S6: Service use over 1-year follow-up

Service (unit)	Intervention				Control			
	n	Mean (SD)	Range	% using	n	Mean (SD)	Range	% using
**Hospital**								
Inpatient - Mental health (nights)	3077	0·04 (0·48)	0 - 12	1%	3005	0·03 (0·51)	0 - 16	1%
Inpatient - Injury (nights)	3085	0·21 (1·29)	0 - 22	5%	3013	0·19 (1·12)	0 - 16	5%
Inpatient - Other (nights)	3063	0·10 (0·86)	0 - 16	2%	2986	0·09 (0·73)	0 - 17	2%
Outpatient - Mental health (appointments)	3007	0·33 (1·96)	0 - 29	5%	2949	0·29 (2·19)	0 - 58	4%
Outpatient - Injury (appointments)	3007	1·38 (4·61)	0 - 85	21%	2911	1·34 (4·61)	0 - 123	21%
Outpatient - Other (appointments)	2937	0·97 (3·09)	0 - 59	18%	2867	0·93 (3·23)	0 - 57	17%
A&E (attendances)	3019	0·74 (2·11)	0 - 31	18%	2943	0·69 (2·13)	0 - 28	17%
Ambulance (attendances)	3120	0·09 (0·67)	0 - 15	3%	3082	0·07 (0·55)	0 - 12	2%
**Community and social care**								
GP	2879	2·29 (4·05)	0 - 47	42%	2829	2·26 (4·26)	0 - 70	43%
Nurse/midwife	3091	0·31 (6·59)	0 - 292	4%	3051	0·17 (1·24)	0 - 36	4%
CAMHS	3045	0·35 (2·53)	0 - 40	4%	3010	0·29 (2·08)	0 - 31	3%
Social worker	3057	0·23 (1·88)	0 - 46	3%	3021	0·16 (1·57)	0 - 42	2%
Pharmacist	2995	0·83 (2·28)	0 - 37	20%	2945	0·89 (2·65)	0 - 43	20%
School nurse	3022	0·97 (3·89)	0 - 112	18%	2966	0·93 (2·96)	0 - 54	19%
Education psychologist	3045	0·50 (4·07)	0 - 172	5%	3015	0·57 (3·85)	0 - 72	5%
Speech and language therapist	3115	0·04 (0·87)	0 - 31	0%	3063	0·05 (0·95)	0 - 36	1%
Counselling	3032	0·56 (3·21)	0 - 49	5%	2993	0·61 (3·83)	0 - 73	5%
Other therapy	3067	0·41 (2·78)	0 - 81	5%	3009	0·42 (2·70)	0 - 65	5%
Helpline	3079	0·27 (4·41)	0 - 213	2%	3030	0·22 (3·56)	0 - 167	2%
**Accommodation**								
Foster care	3136	0·08 (3·04)	0 - 145	<1%	3101	0·01 (0·16)	0 - 9	<1%
Residential care	3143	0·01 (0·33)	0 - 12	<1%	3095	0·02 (0·56)	0 - 28	<1%
Respite care	3128	0·03 (1·13)	0 - 60	<1%	3091	0·04 (0·92)	0 - 33	<1%
**Teaching support**								
Teaching support - some days	2899	-	-	14%	2820	-	-	14%
Teaching support - everyday	2552	-	-	2%	2469	-	-	2%

Kuyken W, *et al. Evid Based Ment Health* 2022; 25:99–109. doi: 10.1136/ebmental-2021-300396

The use of prescribed medication for mental health problems is summarised in Supplementary Table S7. Medication use was similar across trial arms, with 3% of participants using medication for anxiety and depression or eating disorders, 2% using medication for ADHD and 1% using medication for Tics/Tourette's.

Medication for psychosis was reported as most used in both trial arms (5% in intervention and 4% in control). However, this is much higher than what is reported in the literature (antipsychotics were prescribed to 1.74% pupils with intellectual disabilities and 0.12% without) (Henderson et al., 2021). We therefore assessed the impact of excluding medication costs in post-hoc sensitivity analyses (Supplementary Table S10; scenario #9).

Supplementary Table S7: Use of medication over 1-year follow-up

**Medication for:**	**For example:**	**Intervention**		**Control**	
		**n**	**% using**	**n**	**% using**
Anxiety and depression, eating disorders	Fluoxetine, Prozac<br>Sertraline, Lustral<br>Escitalopram, Cipralex<br>Citalopram, Cimpramil<br>Fluvoxamine, Faverin<br>Mirtazapine, Zispin<br>Venlafaxine, Efexor,<br>Atomoxetine	3034	3%	3003	3%
ADHD	Ritalin, Methylphenidate, Equasym<br>Conerta XL, Medikinet XL<br>Dexamphetamine, Dexedrine<br>Atomoxetine, Strattera	3099	2%	3059	2%
Sleep disorders	Dexamphetamine, Dexedrine	3087	2%	3054	3%
Tic/Tourette's	Clonidine, Catapres	3112	1%	3076	1%
Psychosis	Haloperidol	3066	5%	3034	4%

Kuyken W, *et al. Evid Based Ment Health* 2022; 25:99–109. doi: 10.1136/ebmental-2021-300396

Intervention costs

The average cost of delivering SBMT was £1,906·54 per teacher or £73·85 per pupil. A breakdown of the intervention cost is provided in Supplementary Table S8. Total average cost was £176·50 per teacher for phase 1 (self-mindfulness training) and £1,730·04 per teacher for phase 2 (syllabus training).

Supplementary Table S8: Summary of intervention cost

Item	Mean cost
**Phase 1: Self mindfulness training cost per teacher**	
Training	£168·12
Materials	£8·38
**Phase 2: Syllabus training cost per teacher**	
Training	£750
Supply cover	£435·42
Teacher subsistence	£544·62
**Overall**	
Total cost per teacher	£1,906·54
Total cost per pupil	£73·85

Costs and outcomes

Data used in the economic analyses, including costs, CHU9D utilities and QALYs, and the three co-primary clinical outcome measures, are reported in Supplementary Table S9. Total mean costs and QALYs were higher in the intervention arm (mean costs £1333·57, SD=£2389·40; mean QALYs 0·871, SD=0·130) compared to the control arm (mean costs £1290·79, SD=£1379·13; mean QALYs 0·847, SD=0·131). However, differences between trial arms in both costs and QALYs were small in adjusted analyses (adjusted mean difference in cost £6·84, 95% CI -£128·04 to £141·72; adjusted mean difference in QALYs 0·012, 95% CI -0·015 to 0·038). Differences between trial arms in all three primary outcome measures were also small, with better outcomes (lower scores) reported in the control arm compared to the intervention arm.

Kuyken W, *et al. Evid Based Ment Health* 2022; 25:99–109. doi: 10.1136/ebmental-2021-300396

Supplementary Table S9: Mean costs per pupil and economic outcomes over 1-year follow-up

	Intervention (I) mean (SD)	Control (C) mean (SD)	Unadjusted mean difference	Adjusted mean difference (I-C)		
				estimate	95% CI	p-value
**Costs (£)**						
Pre-intervention	360·28 (1242·25)	378·90 (1444·81)	-18·62	-18·81	-84·96 to 47·33	0·57
Intervention	70·73 (21·66)	0·00 (0·00)	70·73	71·61	64·84 to 78·38	<0·0001
Hospital services	601·55 (1569·74)	636·51 (1660·74)	-35·73	-36·70	-120·64 to 47·24	0·39
Community health and social care services	377·92 (1014·23)	636·51 (1660·74)	-6·14	-21·97	-73·21 to 29·27	0·40
Medication	17·18 (76·98)	18·81 (83·44)	-2·74	-2·08	-6·94 to 2·78	0·40
Accommodation services	23·54 (458·70)	18·03 (426·27)	5·51	5·30	-14·96 to 25·57	0·61
Teaching support	242·59 (810·92)	232·54 (755·87)	3·72	-6·33	-53·35 to 40·69	0·79
Total	1333·57 (2389·42)	1290·79 (1379·13)	34·90	6·84	-128·04 to 141·72	0·92
**Health-related quality of life (CHU9D)**						
Utility score pre-intervention	0·838 (0·118)	0·837 (0·116)				
Utility score post-intervention	0·825 (0·127)	0·828 (0·124)				
Utility score 1-year follow-up	0·824 (0·129)	0·823 (0·129)				
Total QALYs	0·871 (0·130)	0·847 (0·131)	0·017	0·012	-0·015 to 0·038	0·39
**Primary outcomes**						
Depression (CES-D) [a]	17·34 (11·95)	16·94 (11·95)	0·34	0·27	-0·62 to 0·70	0·91
Social/emotional/behavioural functioning (SDQ) [a]	13·46 (6·86)	13·19 (6·87)	0·27	0·15	-0·17 to 0·50	0·33
Well-being (WEMWBS) [a]	47·39 (9·86)	47·35 (9·87)	0·02	0·02	-0·34 to 0·69	0·51

[a] Lower scores indicate better outcomes

Kuyken W, *et al. Evid Based Ment Health* 2022; 25:99–109. doi: 10.1136/ebmental-2021-300396

Cost-effectiveness analyses

Incremental costs and effects over 1-year follow-up for all analyses are presented in Supplementary Table S10.

The main cost-utility analysis using QALYs as the outcome of interest (scenario #1) suggests that SBMT has a higher probability (83%;) of being cost-effective at the willingness-to-pay threshold range used by NICE (£20,000-£30,000 per QALY) compared to control. All sensitivity tests of the main cost-utility analysis (three pre-specified and outlined in the main paper and two post-hoc and outlined in this supplement) support this suggestion that SBMT has a higher probability of being cost-effective (probability >50%) than control for willingness-to-pay thresholds ranging from £20,000-£30,000 per QALY (Supplementary Figure S2). Secondary cost-effectiveness analyses, using the primary clinical outcomes as the measure of effect, suggest that SBMT has a lower probability of being cost-effective (<40%; Supplementary Figure S1) than control for all willingness-to-pay thresholds.

Kuyken W, *et al. Evid Based Ment Health* 2022; 25:99–109. doi: 10.1136/ebmental-2021-300396

Supplementary Table S10: Incremental costs and effects over 1-year follow-up (joint effects)

Scenario		Incremental cost		Incremental effect		Net monetary benefit	
		Mean	SE	Mean	SE	WTP=£20,000	WTP=£30,000
**Main analyses**							
1	Base-case using QALY	31·21	72·42	0·0135	0·0135	239·07	374·21
2	Base-case using CES-D	37·88	72·28	-0·1059	0·3391	-2155·97	-3215·02
3	Base-case using SDQ	38·82	71·73	-0·1943	0·1744	-3924·21	-5866·91
4	Base-case using WEMWBS	38·29	71·95	-0·1474	0·2636	-2985·55	-4459·18
**Sensitivity analyses**							
5	Complete cases	106·58	77·06	0·0098	0·0132	89·17	187·05
6	Health and social care perspective	26·04	60·39	0·0135	0·0135	244·76	380·16
7	Mental health services perspective	49·66	29·16	0·0115	0·0135	180·51	295·59
8	Excluding late follow-ups	32·33	74·47	0·0155	0·0115	278·17	433·43
9	Excluding medication	31·53	70·99	0·0137	0·0135	243·25	380·64

Kuyken W, *et al. Evid Based Ment Health* 2022; 25:99–109. doi: 10.1136/ebmental-2021-300396

Supplementary Figure S1: Cost-effectiveness acceptability curves for main economic analyses (scenarios 1-4)

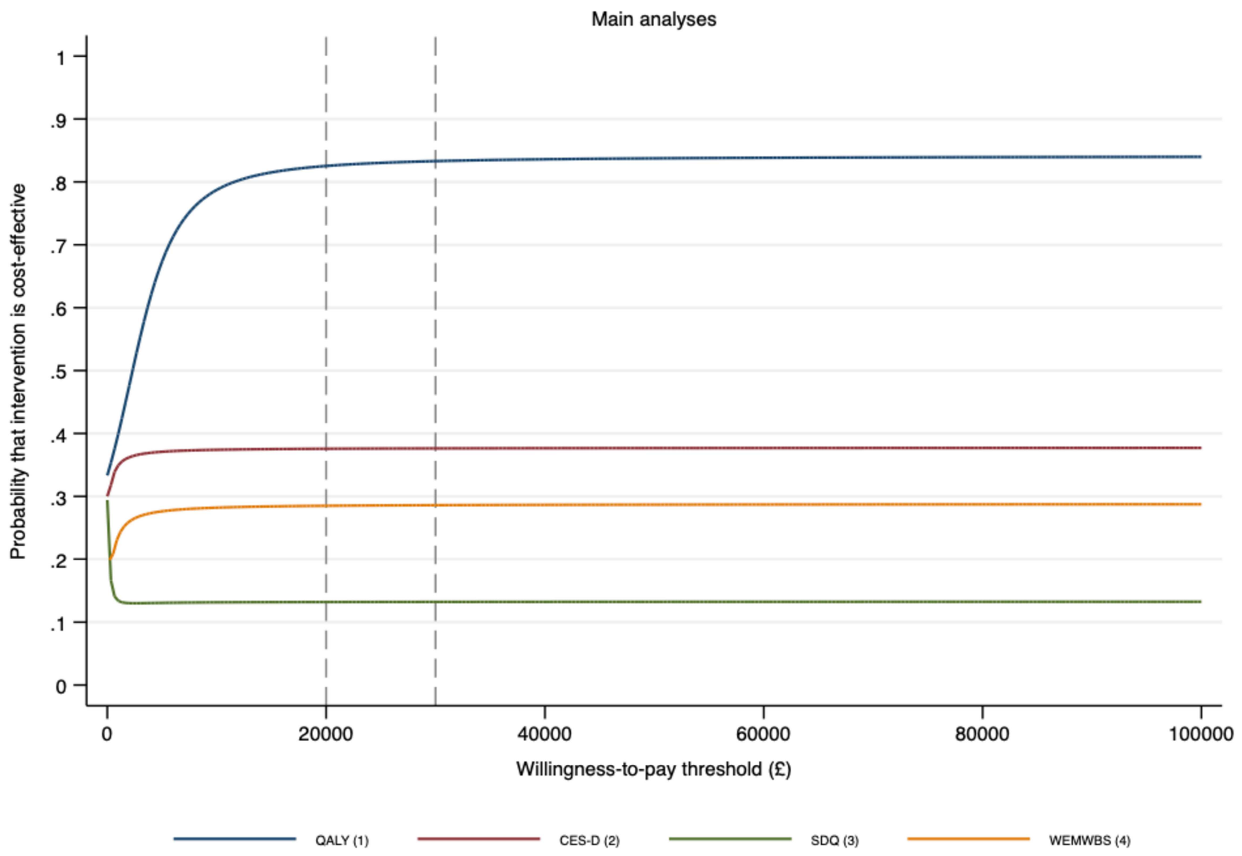

Kuyken W, *et al. Evid Based Ment Health* 2022; 25:99–109. doi: 10.1136/ebmental-2021-300396

Supplementary Figure S2: Cost-effectiveness acceptability curves for sensitivity analyses (scenarios 5-9)

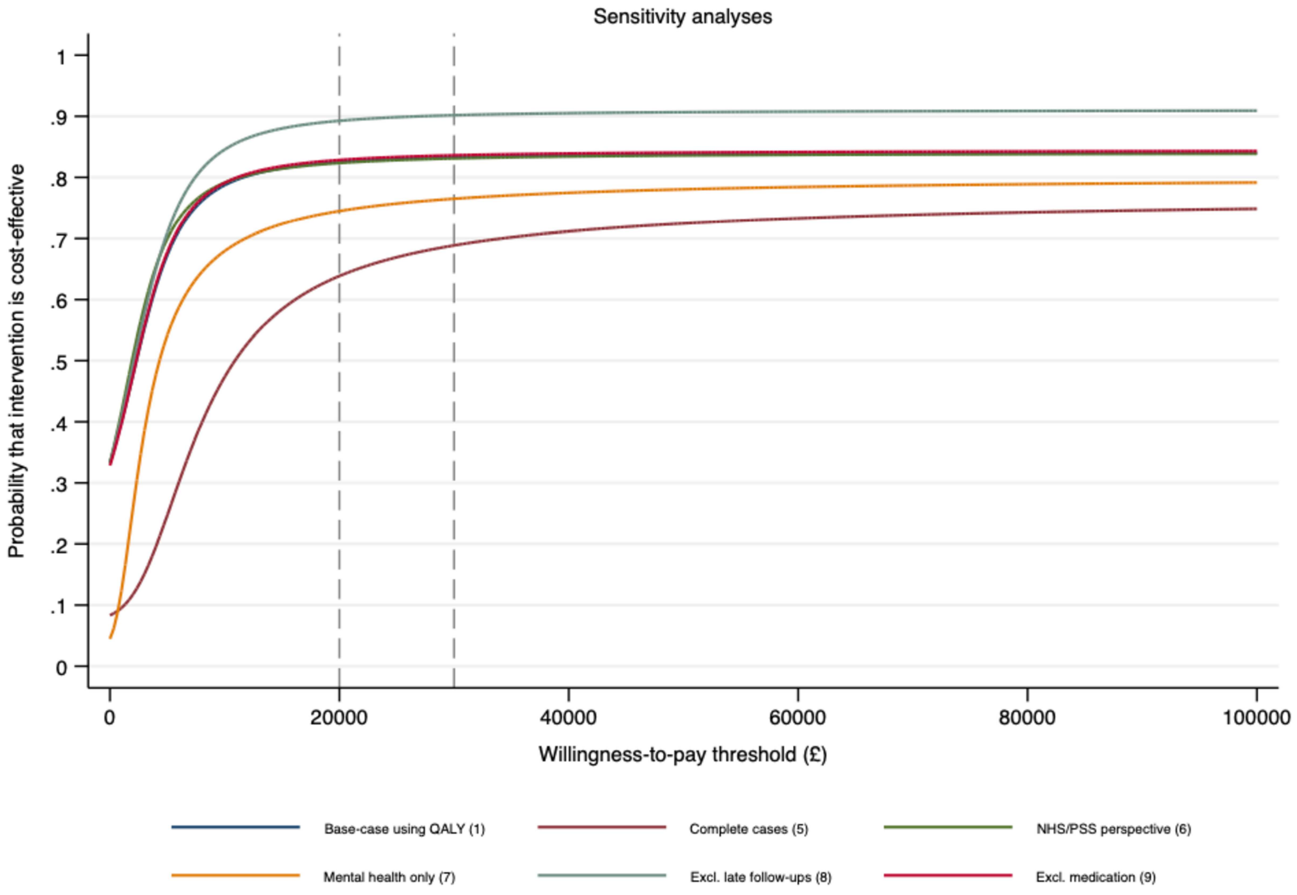

References

Association of Alaska School Boards. School climate and connectedness survey [measurement instrument]. Juneau: Association of Alaska School Boards; 2015.

Briere FN, Pascal S, Dupere V, Janosz M. School environment and adolescent depressive symptoms: A multi-level longitudinal study. Pediatrics. 2013.

Byford S, Barrett B, Roberts C, Wilkinson P, Dubicka B, Kelvin RG, et al. Cost-effectiveness of selective serotonin reuptake inhibitors and routine specialist care with and without cognitive-behavioural therapy in adolescents with major depression. British Journal of Psychiatry. 2007;191:521-7.

Clarke A, Friede T, Putz R, Ashdown J, Martin S, Blake A, et al. Warwick-Edinburgh Mental Well-being Scale (WEMWBS): Validated for teenage school students in England and Scotland. A mixed methods assessment. BMC Public Health. 2011;11

Clarke, A., Friede, T., Putz, R., Ashdown, J., Martin, S., Blake, A., Adi, Y., Parkinson, J., Flynn, P., Platt, S., & Stewart-Brown, S. (2011). Warwick-Edinburgh Mental Well-being Scale (WEMWBS): validated for teenage school students in England and Scotland. A mixed methods assessment. BMC public health, 11, 487. https://doi.org/10.1186/1471-2458-11-487

Crane C, Ganguli P, Ball S, Taylor L, Blakemore S-J, Byford S, et al. Training School Teachers to Deliver a Mindfulness Program: Exploring Scalability, Acceptability, Effectiveness, and Cost-effectiveness. Global advances in health and medicine. 2020; 9: 2164956120964738.

Crane, R.S., Koerbel, L., Sansom, S., Yiangou, A. (2020) Assessing Mindfulness-Based Teaching Competence: Good Practice Guidance, Global advances in Health and Medicine, https://doi.org/10.1177/2164956120973627

Curtis L, Burns A. Unit Costs of Health and Social Care. Personal Social Services Research Unit, University of Kent, Canterbury; 2019.

de Bruin EI, Zijlstra BJH, Bögels SM. The Meaning of Mindfulness in Children and Adolescents: Further Validation of the Child and Adolescent Mindfulness Measure (CAMM) in Two Independent Samples from The Netherlands. Mindfulness. 2013; 5(4):422-30.

Department of Education. Personal, social, health and economic (PSHE) education: A mapping study of the prevalent models of delivery and their effectiveness. London; 2010. Contract No.: DFE-RR080.

Department of Education. Schools, pupils and their characteristics: January 2020 London, UK2020 [Available from: https://www.gov.uk/government/statistics/schools-pupils-and-their-characteristics-january-2020.

Department of Health (DH). NHS Reference Costs 2018-19. London DH; 2019.

Fenwick E, Byford S. A guide to cost-effectiveness acceptability curves. Br J Psychiatry. 2005; 187:106-8.

Ford T, Esposti MD, Crane C, Taylor L, Montero-Marin J, Blakemore S-J, et al. The Role of Schools in Early Adolescents' Mental Health: Findings From the MYRIAD Study. Journal of the American Academy of Child and Adolescent Psychiatry. 2021.

Ford T, Hayes R, Byford S, Edwards V, Fletcher M, Logan S, et al. The effectiveness and cost-effectiveness of the Incredible Years® Teacher Classroom Management programme in primary school children: results of the STARS cluster randomised controlled trial. Psychological Medicine. 2018: 1-15.

Furber G, Segal L. The validity of the Child Health Utility instrument (CHU9D) as a routine outcome measure for use in child and adolescent mental health services. Health and quality of life outcomes. 2015; 13:22.

Gioia, G. A., Isquith, P. K., Guy, S. C., Kenworthy, L. (2015). Behavior Rating Inventory of Executive Function®, Second Edition (BRIEF®2). Lutz, FL: PAR Inc.

Goodman R, Renfrew D, Mullick M. Predicting type of psychiatric disorder from Strengths and Difficulties Questionnaire (SDQ) scores in child mental health clinics in London and Dhaka. Eur Child Adolesc Psychiatry. 2000; 9(2):129-34.

Goodman R. Psychometric properties of the Strengths and Difficulties Questionnaire. J Am Acad Child Adolesc Psychiatry. 2001; 40:1337–45.

Greco, L.A., Baer, R.A., & Smith, G. (2011). Assessing mindfulness in children and adolescents:Development and validation of the child and adolescent mindfulness measure (CAMM).Psychological Assessment, 23. 606-614.

Henderson A, Kinnear D, Fleming M, Stanley B, Greenlaw N, Young-Southward G, et al. Antipsychotic and antidepressant prescribing for 704 297 children and young people with and without intellectual disabilities: record linkage study. The British Journal of Psychiatry. 2021; 218(1):58-62.

Huppert, F. A., & Johnson, D. M. (2010). A controlled trial of mindfulness training in schools: The importance of practice for an impact on well-being. Journal of Positive Psychology, 5(4), 264-274. https://doi.org/Doi 10.1080/17439761003794148

Kuby, A.K., McLean, N. & Allen, K. Validation of the Child and Adolescent Mindfulness Measure (CAMM) with Non-Clinical Adolescents. Mindfulness 6, 1448–1455 (2015). https://doi.org/10.1007/s12671-015-0418-3.

Kuyken W, Bernard P, Baer R. Mindfulness-based Cognitive Therapy for Life Teacher's Guide. Manuscript in preparation: Guilford Press; 2019.

Kuyken, W., Weare, K., Ukoumunne, O. C., Vicary, R., Motton, N., Burnett, R., Cullen, C., Hennelly, S., & Huppert, F. (2013). Effectiveness of the Mindfulness in Schools Programme: non-randomised controlled feasibility study. British Journal of Psychiatry, 203(2), 126-131. https://doi.org/Doi 10.1192/Bjp.Bp.113.126649

Madge, N., Hewitt, A., Hawton, K., de Wilde, E. J., Corcoran, P., Fekete, S., van Heeringen, K., De Leo, D., & Ystgaard, M. (2008). Deliberate self-harm within an international community sample of young people: comparative findings from the Child & Adolescent Self-harm in Europe (CASE) Study. Journal of child psychology and psychiatry, and allied disciplines, 49(6), 667–677. https://doi.org/10.1111/j.1469-7610.2008.01879.x.

Mindfulness in Schools Project. (2016). How to teach .b. Teachers Notes. Curriculum available from: http://mindfulnessinschools.org/.

Montero-Marin J, Taylor L, Crane C, Greenberg MT, Ford TJ, Williams JMG, et al. Teachers Finding Peace in a Frantic World': An experimental study of self-taught and instructor-led mindfulness program formats on acceptability, effectiveness and mechanisms. Journal of Educational Psychology, 2021. https://doi.org/10.1037/edu0000542

National Education Union. Support staff pay and conditions 2019.

NHS Business Services Authority Statistics. Prescription Cost Analysis - England 2019 2019.

NSPCC. NSPCC - How your money is spent 2021. Samaritans - How your money can help us; 2021.

Radloff LS. The Use of the Center for Epidemiologic Studies Depression Scale in Adolescents and Young-Adults. Journal of Youth and Adolescence. 1991;20(2):149-66.
Royal Pharmaceutical Society of Great Britain. British National Formulary for Children 2019.

Rushton, J. L., Forcier, M., & Schectman, R. M. (2002). Epidemiology of depressive symptoms in the National Longitudinal Study of Adolescent Health. Journal of the American Academy of Child and Adolescent Psychiatry, 41(2), 199–205. https://doi.org/10.1097/00004583-200202000-00014.

Segal ZV, Williams JMG, Teasdale JD. Mindfulness-based cognitive therapy for depression. Second edition ed. New York: Guilford Press; 2013.

Stevens K, Ratcliffe J. Measuring and valuing health benefits for economic evaluation in adolescence: an assessment of the practicality and validity of the child health utility 9D in the Australian adolescent population. Value Health. 2012;15:1092–9.

Stevens K. Assessing the performance of a new generic measure of health-related quality of life for children and refining it for use in health state valuation. Appl Health Econ Health Policy. 2011;9:157–69.

Stevens K. The Development of a Preference Based Paediatric Health Related Quality of Life Measure foruse in Economic Evaluation. PhD Thesis 2008. The University of Sheffield.

Stinnett AA, Mullahy J. Net health benefits: a new framework for the analysis of uncertainty in cost-effectiveness analysis. Med Decis Making. 1998; 18(2 Suppl):S68-80.

Tennant R, Hiller L, Fishwick R, Platt S, Joseph S, Weich S, Parkinson J, Secker J, Stewart-Brown S. The Warwick-Edinburgh mental well-being scale (WEMWBS): development and UK validation. Health Qual Life Outcomes. 2007; 5:63.

Thompson SG, Barber JA. How should cost data in pragmatic randomised trials be analysed? BMJ. 2000; 320(7243):1197-200.

Tudor, K., Raja, A., Maloney, S., Baer, R., Blakemore, S., Byford, S., … Montero-Marin, J. (2021, June 17). Universal mindfulness training in schools for adolescents: A scoping review and conceptual model of moderators, mediators and implementation factors. https://doi.org/10.31219/osf.io/j2qz7

Weiss DC, Chorpita BF. Revised children's anxiety and depression scale: user's guide. 2011. http://www.childfirst.ucla.edu/
